# Evaluating the limitations of Bayesian metabolic control analysis

**DOI:** 10.1371/journal.pcbi.1012987

**Published:** 2026-01-16

**Authors:** Janis Shin, James M. Carothers, Herbert M. Sauro

**Affiliations:** 1 Molecular Engineering & Sciences Institute, Center for Synthetic Biology, University of Washington, Seattle, Washington, United States of America; 2 Department of Chemical Engineering, Center for Synthetic Biology, University of Washington, Seattle, Washington, United States of America; 3 Department of Bioengineering, Center for Synthetic Biology, University of Washington, Seattle, Washington, United States of America; Inria Saclay: Inria Centre de Recherche Saclay-Ile-de-France, FRANCE

## Abstract

Bayesian Metabolic Control Analysis (BMCA) is a promising framework for inferring metabolic control coefficients in data-limited scenarios, combining Bayesian inference with linear-logarithmic (lin-log) rate laws. These metabolic control coefficients quantify how changes in enzyme activities affect steady-state fluxes and metabolite concentrations across a metabolic network. However, its predictive accuracy and limitations remain underexplored. This study systematically evaluates BMCA’s ability to infer elasticity values, flux control coefficients (FCC), and concentration control coefficients (CCC) under varying data availability conditions using three synthetic metabolic network models. We demonstrate that BMCA predictions are highly dependent on the inclusion of flux and enzyme concentration data, with the omission of these datasets leading to severe inaccuracies. In our synthetic, enzyme-perturbation datasets, external metabolite concentrations had minimal impact and, in some cases, their exclusion improved predictions; when external-nutrient perturbations were introduced and those concentrations were observed, gains were at most modest. Additionally, we find that posterior estimation with both ADVI and HMC can underestimate large-magnitude elasticities in our synthetic settings, with ADVI showing somewhat higher variance under strong up-regulation; thus, recovering |elasticity| ≳1.5 remains challenging regardless of the inference engine. ADVI also fails to accurately infer allosteric interactions, even when regulatory effects are strong. While BMCA maintains reasonable accuracy in partially recovering the rankings of the highest FCC values, its estimates of absolute values remain constrained by prior assumptions and data limitations. Our findings reveal the BMCA algorithm’s strengths and weaknesses, providing guidance on its application in metabolic engineering, and highlighting the need for methodological refinements to enhance its predictive capabilities.

## Author summary

Bayesian Metabolic Control Analysis (BMCA) has emerged as a promising framework for inferring metabolic control coefficients in data-limited scenarios by integrating Bayesian inference with linear-logarithm rate laws. However, its predictive accuracy and limitations remain underexplored. This study systematically evaluates BMCA’s ability to infer elasticity values, flux control coefficients (FCCs), and concentration control coefficients (CCCs) under varying data availability conditions using three synthetic metabolic network models. Our findings highlight the strengths and weaknesses of BMCA, guiding its application in metabolic engineering and emphasizing the need for methodological refinements.

## Introduction

Much research has focused on optimizing the process of engineering metabolic pathways to biomanufacture chemicals sustainably [1,2]. Many groups have demonstrated that it is possible to design alternate routes for carbon molecules through existing metabolic networks in organisms to produce desired chemical products, such as amino acids, oleochemicals, and isoprenoids [3–5]. To meet the demand for biomanufactured chemicals, the process of reengineering endogenous metabolic networks needs to be accelerated.

One approach for reengineering metabolic pathways is to build a kinetic model to identify control points in a metabolic pathway, which can then be targeted to shift metabolic rates in favor of producing the desired product [6–8]. To analyze control distribution within a pathway, some mechanistic models incorporate metabolic control analysis (MCA) [7], a mathematical framework that quantifies how control is distributed among individual enzymes. Using MCA, one can calculate elasticities, which measure how enzyme rates are affected locally by changes in substrates, products, and other effectors. MCA then defines two system-based measurements called flux and concentration control coefficients which are functions of elasticities [9,10]. The flux control coefficient (FCC) values describe how a change in the concentration of an enzyme can affect fluxes through all the reactions in the pathway. Similarly, the concentration control coefficient (CCC) values describe how a change in concentration of an enzyme can affect the concentrations of metabolites in the pathway. The FCC values with the greatest magnitudes represent the most promising enzyme targets.

While knowledge of the metabolic network structure, allosteric interactions, kinetic parameters, metabolite concentrations, and flux values is valuable for determining control coefficients using MCA, many kinetic parameters within a metabolic pathway remain unknown, necessitating empirical approaches for estimating these coefficients. One method involves measuring the resulting fluxes and concentrations from direct perturbations to the enzyme or perturbing enzymes using inhibitors [11]. Unfortunately, this method does not easily scale. If the metabolite concentrations are known, control coefficients can also be estimated based on elasticities using various techniques, such as conducting simulations to build models (primarily for smaller pathway segments) and performing modulation experiments to assess changes in metabolite levels after manipulating upstream or downstream components. While these methods can provide valuable insights, they do not easily scale to whole-cell studies.

Bayesian Metabolic Control Analysis (BMCA) is an approach to a mechanistic model that combines Bayesian inference with linear-logarithmic (lin-log) rate laws and whole-cell physiological data to infer elasticity values, which can subsequently be used to estimate control coefficients [12–14]. This approach is particularly advantageous in data-limited situations, as Bayesian inference allows the incorporation of prior information about a system into the analysis. By leveraging existing information, BMCA enhances the statistical power to detect meaningful associations and effects. In practice, to apply Bayesian inference, one needs a prior, a likelihood function, and observed data, if available. BMCA models each reaction using lin-log kinetics [12,13,15], which are simplified and approximate rate laws made using a steady-state assumption that use elasticity values as parameters—the lin-log rate laws function as part of the likelihood in the Bayesian inference. Although lin-log rate laws are an approximation, they appear to be robust to large perturbations to the system [16]. If available, whole-cell data (such as flux values, enzyme concentrations, and metabolite concentrations) can be input into the Bayesian framework as observed data. Through the combination of mechanistic understanding and prior information, BMCA generates posterior probability distributions for the elasticities, from which control coefficients are subsequently calculated.

This paper considers the following questions: What type of physiological data and how much data is necessary for BMCA to make helpful predictions? How effective is BMCA at predicting allosteric interactions? We address these questions using three synthetic models from which we establish ground truth values such as elasticities and control coefficients. We evaluated how accurately BMCA inferred elasticity values across all the reactions in the tested network topologies and FCC rank orders for the reaction directly involved in producing a specific output.

We found that flux data, enzyme concentration data, and internal metabolite concentration data were critical for determining elasticity values and concentration control coefficients, whereas flux data and enzyme concentration data were more critical for determining flux control coefficients. Contrary to previous claims about the BMCA algorithm’s ability to detect implicit allosteric relationships [12], our work demonstrated that BMCA failed to detect allosteric relationships for the three models we investigated, even if the allostery was strong. Additionally, the BMCA prior predictions and posterior predictions for the top ten reactions with the highest FCCs were only marginally different. These insights can provide future guidance for researchers when using BMCA and help clarify the predictive limits of BMCA.

### Building the test models and simulated datasets

**Fig 1:**
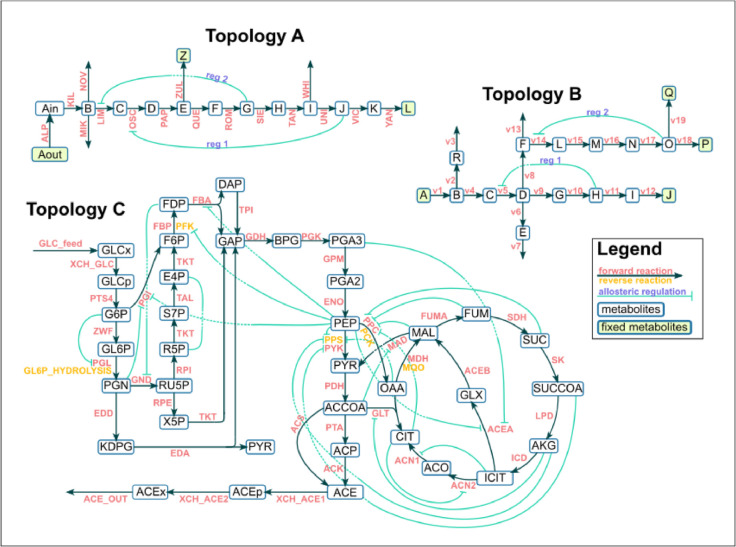
Three synthetic networks used to benchmark BMCA. A) TopA, B) TopB, C) TopC, adapted from Millard et al. [18]. Cyan lines describe the allosteric regulation. For TopC, only negative allosteric regulation is shown.

Three different model topologies were constructed (Fig 1), two of which included variations incorporating allosteric regulators, for a total of seven model variations. Topology A (TopA) is a linear chain, with several branch points. TopA has three total variations of possible allosteric regulation: one without any, one with metabolite J regulating the reaction labeled OSC, and one with metabolite J regulating reaction OSC in addition to metabolite G regulating reaction LIM. For all TopA models, reaction YAN was optimized. Topology B (TopB) exhibits more branching than TopA and also has three total variations of possible allosteric regulation: one without any, one with metabolite H regulating reaction v5, and one with metabolite H regulating reaction v5 in addition to metabolite O regulating reaction v14. Topology C (TopC) is loosely based on E. coli central metabolism and contains not only conserved moieties (which TopA and B do not have), but also a cycle in the form of the Krebs cycle. Summaries of ground truth coefficient values for all three networks can be found in (Table 1).

**Table 1:** Summaries of ground truth coefficient value ranges for all three networks and their allosteric variations.

Network	Coefficient Type	Condition	Min. Value	Max. Value
TopA	Elasticities	noReg	–1.43	1.45
		Reg1	–1.58	1.85
		Reg2	–1.70	1.71
	CCCs	noReg	–1.36	0.84
		Reg1	–1.14	0.78
		Reg2	–0.98	0.74
	FCCs	noReg	–0.83	0.96
		Reg1	–0.78	0.96
		Reg2	–0.79	0.95
TopB	Elasticities	noReg	–10.94	11.74
		Reg1	–14.95	15.75
		Reg2	–22.82	23.64
	CCCs	noReg	–1.70	2.53
		Reg1	–1.60	1.87
		Reg2	–1.60	1.86
	FCCs	noReg	–0.86	1.15
		Reg1	–0.96	1.03
		Reg2	–0.96	1.02
TopC	Elasticities	-	–1.885	1.885
	CCCs	-	–2.01	2.07
	FCCs	-	–3.21	5.93

## Materials and methods

### Experimental setup overview

**Fig 2:**
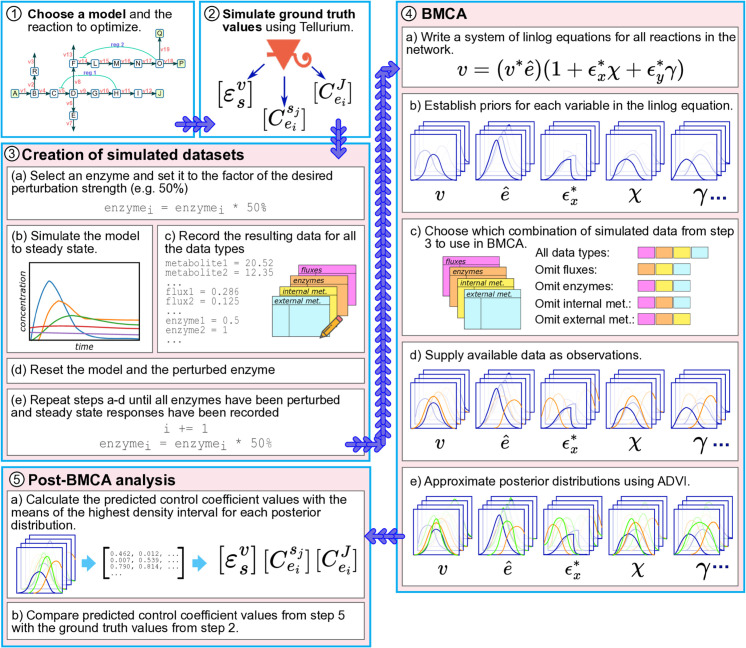
Overview of BMCA benchmarking experiments. Synthetic models are simulated using tellurium (16) to produce datasets of fluxes and concentrations of enzymes, internal metabolites, and external metabolites. The ground truth FCC values and elasticities are also calculated from the simulation. For each model variation, five parallel BMCA runs take place: one without any omitted data and one each of omitting one data type. Each BMCA run results in a set of posterior distributions for elasticities. The HDI for each posterior distribution is computed, resulting in a single value for each elasticity. These elasticity sets are compared with ground truth elasticity values and then used to calculate control coefficient values which are subsequently compared with the ground truth control coefficients.

In our study, we evaluated each model variation using the following experimental setup (Fig 2). Ground truth values for the elasticities and control coefficients were calculated using numerical differentiation in Tellurium. Specifically, we applied a 5-point backward finite difference formula to steady-state simulations of the network models. Perturbations were made to enzyme concentrations, and system responses were computed by re-simulating the steady state after each perturbation. The models were then simulated to steady state and four types of datasets were generated: flux values for each reaction and concentrations for each enzyme, internal metabolite, and external metabolite. The choice of data types to include or omit from the BMCA algorithm as well as the presence of the allosteric regulators was varied. Each variation was used as the observational data for the BMCA algorithm. The variation where no data was omitted represented a best-case scenario for the study. We then executed BMCA multiple times, omitting one data type at a time to assess the contribution of each type to the predictive capability of the algorithm. Each run produced posterior distributions for all elasticities, from which we calculated the mean of the highest density interval (HDI). These means were compared against the ground truth values, and FCCs were estimated from the elasticity HDI means. Finally, we ranked the reactions based on both ground truth and predicted FCC values for comparison.

### Construction of test models

TopA was based on a yeast glycolysis model by Teusink et al. (BIOMD0000000064) [11,17]. The ATP and NADH assignment rules were removed and the enzyme parameters were changed to values so that the reactions were not at equilibrium. TopA has 14 metabolites and 16 reactions. Reaction YAN is the reaction being optimized in all the BMCA analyses. TopB has 17 metabolites and 19 reactions. Reaction v19 is the reaction being optimized in all the BMCA analyses. TopC was based on an E. coli model by Millard et al. (MODEL1505110000) [18] that includes the pentose phosphate pathway and the TCA cycle. It has 64 metabolites and 68 reactions with the flux through reaction ACE_OUT to be optimized.

### Generating simulated data

Since the lin-log rate law used in BMCA assumes the system is at steady state, the method requires input fluxes that satisfy the steady-state condition: that the product of the stoichiometric matrix and the reference flux vector must be zero. Thus, to generate simulated data, models and their variations were run to steady state using Tellurium [19,20] for the following enzyme perturbation levels: 10%, 20%, 30%, 40%, 50%, 150%, 300%, 500%, 700%, and 1000%. At steady state, the relative enzyme concentrations, as well as the absolute metabolite concentrations and reaction fluxes, were recorded. Each enzyme was then perturbed by a fixed amount (e.g., 10% of its original level), and the system was re-simulated to record the new steady-state metabolite concentrations, reaction fluxes, and relative enzyme concentrations. After each perturbation, the model was reset to ensure that the data reflected the response to a single enzyme perturbation only. In some cases, perturbations pushed the system into parameter regimes where Tellurium failed to compute a steady state because none existed. In such cases, data were not generated, and the corresponding perturbations were excluded to ensure that only inputs consistent with steady state were used for BMCA.

The aforementioned results were produced on datasets where only the enzymes were perturbed. Preliminary experiments indicated that incorporating perturbed external metabolite data into the perturbation datasets showed modest improvement on BMCA predictions within the tested perturbation ranges (Fig A in S1 Text). Therefore, we proceeded to only use enzyme perturbed data in the perturbation datasets moving forward.

### Setting up BMCA

When all data are provided, the lin-log model is formulated as:

$$\hat{v}=\hat{e}(1_{n}+\varepsilon_{x}\chi +\varepsilon_{y}\gamma ),$$ (1)

where $\hat{v}=\frac{v}{v^{*}}$, $\hat{e}=\frac{e}{e^{*}}$, $\chi =log(\frac{x}{x^{*}})$, and $\gamma =log(\frac{y}{y^{*}})$. *v* represents the flux data, *e* the enzyme concentrations, *x* the internal metabolite concentrations, and *y* the external metabolite concentrations. The star denotes values at the reference state. Any experiment can be chosen as the reference state; however, because lin-log models are most valid in the vicinity of the reference condition, the chosen state should be representative of the operating regime of interest. For this paper, the reference state was designated as the experiment where none of the enzymes were perturbed, for simplicity.

### Prior distributions and control coefficient determination

The priors for the elasticities ($\varepsilon_{x}$ and $\varepsilon_{y}$) were established as described by St. John et al. [12]. The priors for the other variables are listed below in Table 2. The priors for normalized enzyme concentrations ($\hat{e}$) were centered around 1 for unperturbed states and adjusted accordingly for perturbed states. For the χ and *γ* parameters, we employed broad Normal priors centered at 0 with a standard deviation of 10, reflecting a weakly informative prior belief. Additionally, the flux priors were centered on values predicted by the lin-log model equation (Eq. 1), aligning with the assumption that the lin-log model provides a reasonable approximation of fluxes given the other variables.

**Table 2:** Prior distributions of the BMCA model.

Symbol	Distribution	Center (*μ*)	Other params.
$\varepsilon_{x}$	Skew-normal	Uniform[0.1, 1.1]	*α* = 5
$\varepsilon_{y}$	Laplace	Uniform[0.1, 1.1]	*b* = 0.05
$\hat{e}$	Normal	1	σ=1
χ	Normal	0	σ=10
*γ*	Normal	0	σ=10
*v*	Normal	$\hat{e}(1_{n}+\varepsilon_{x}\chi +\varepsilon_{y}\gamma )$	σ=0.1

The CCCs and FCCs were also calculated using the method described by St. John et al. However, FCC predictions for fluxes in which an enzyme perturbs its own reaction exhibited a consistent deviation of –2. To account for this systematic offset, a correction factor of +2 was applied to these predictions (Fig B in S1 Text).

Bayesian inference is performed using Automatic Differentiation Variational Inference (ADVI), which optimizes the evidence lower bound (ELBO) to approximate the posterior distributions of the model parameters.

When specific data types are omitted, the missing values are treated as unmeasured data, and latent variables are introduced to account for the missing information. In cases where flux or enzyme data are unavailable, the posterior distributions of these latent variables are later used to estimate CCCs and FCCs. If flux data are omitted, only the reference fluxes are supplied to the Bayesian inference model, and the lin-log model is reformulated to solve for enzyme concentrations instead of fluxes as follows:

$$\hat{e}=\hat{v}(1_{n}+\varepsilon_{x}\chi +\varepsilon_{y}\gamma )$$ (2)

## Results

The goal of this paper is to determine which data types are most critical for BMCA predictions, how well BMCA predicts latent allostery from data, and how well BMCA predicts elasticity coefficients, CCCs, and FCCs. If exact values cannot be recovered, we will assess whether the relative rankings of the predicted coefficients align with those of the ground truth, as this may still guide experimental prioritization.

To address these questions, we tested BMCA using our three model topologies and their respective datasets, with outcomes analyzed across different perturbation and allostery levels and proportions of observed data input.

### Benchmarking and validation of BMCA implementation

#### Comparability of v-based and *emll* implementations.

To ensure a valid comparison with previously published results, we reproduced *emll*, the method used by St. John et al. [12]. We used a slightly different implementation of BMCA called “v-based" (since we solve for flux). Both *emll* and the v-based method rely on the lin-log rate law, but they differ in how elasticities are obtained. The *emll* approach substitutes the lin-log expressions into the global steady-state constraint (Nv=0) and solves a single coupled linear system for all elasticities simultaneously, whereas the v-based method retains the per-reaction lin-log form and estimates elasticities individually from observed flux changes.

While the original study used the Wu et al. and Contador models, these lacked full ground truth values for all elasticity and control coefficients. Our synthetic models overcome this limitation and allow for a more rigorous benchmarking of both implementation and inference accuracy. Therefore, we used our own synthetic TopB model to compare both methods.

**Fig 3:**
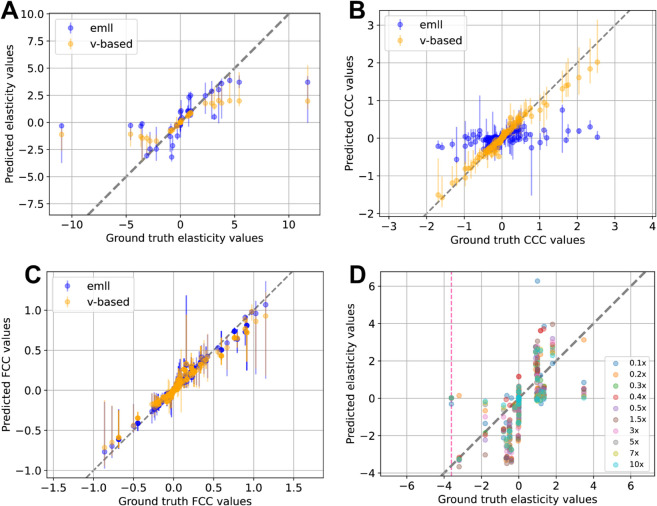
Comparison of BMCA predictions from the emll method and the v-based method on TopB. (A) Elasticities, (B) CCCs, and (C) FCCs predicted by BMCA for TopB using both the *emll* and v-based methods. No data were omitted, and no allosteric regulation was present. Each dot represents the median of the predicted distribution means across ten different enzyme perturbation strengths. Error bars indicate the range of these predictions. (D) Elasticity predictions from the *emll* method for TopA with a strong allosteric regulator (ground truth elasticity = –3.61). The method was applied to ten datasets with varying enzyme perturbation strengths; colored dots indicate which dataset each prediction came from. The gray dashed line marks perfect agreement with ground truth values, and the pink dashed line marks the ground truth elasticity of the regulator.

In comparing the original *emll* method to our v-based implementation, we found that *emll* consistently overestimated elasticity values when the true magnitude was below 4, whereas the v-based method maintained better accuracy for ground truth values below magnitude 2 (Fig 3A). Notably, *emll* failed to recover any CCCs, while the v-based approach produced accurate CCC predictions (Fig 3B). For FCCs, both methods produced comparable estimates, across all the different perturbation strengths (Fig 3C).

Overall, the predictions produced by the v-based implementation closely mirrored those of the original *emll* method for both elasticities and FCCs.

#### Variance and accuracy of ADVI versus HMC.

Having validated the modeling implementation, we next evaluated the impact of inference method on posterior accuracy and variance. To assess whether ADVI adequately captures posterior variance in BMCA predictions, we compared it to Hamiltonian Monte Carlo (HMC), which is known to provide more accurate posterior estimates by avoiding the approximations inherent in variational inference [21]. We used the No-U-Turn Sampler (NUTS) [22] algorithm to implement HMC for a fair comparison with St. John’s previous work. We performed this direct comparison of ADVI and HMC on TopA and TopB model variations that did not have any allosteric regulators.

For the NUTS sampler, we assessed convergence and mixing using standard diagnostics. All parameters from both TopA and B had effective sample sizes greater than 500 and $\hat{r}$ values less than 1.01. No post-warmup divergent transitions were observed.

For TopA, both methods produced nearly identical elasticity predictions in terms of magnitude and variance (Figs C1 and C2 in S1 Text), suggesting that ADVI performs adequately on simpler models. Variance was generally low across both methods. In TopB, ADVI and HMC agreed within the ±1.5 elasticity range, but diverged at higher ground truth magnitudes. HMC accurately recovered elasticities up to magnitude 5, particularly under strong perturbations (≥3x); for weaker perturbation strengths (<3x), it exhibited a capping effect comparable to ADVI’s.

For enzyme knockdown perturbations (0.1x to 0.5x), we observed no consistent bias in variance estimates between HMC and ADVI (Figs C3 and C5 in S1 Text). In contrast, for upregulation perturbations (1.5x to 10x), ADVI consistently reported higher variances than HMC (Figs C4 and C6 in S1 Text). This difference was primarily due to the fact that HMC often produced near-zero or zero variance estimates, whereas ADVI yielded small but nonzero variances. Overall, HMC variance tended to decrease to near-zero as perturbation strength increased. Although ADVI variances were technically higher than those from HMC across both TopA and B, the differences in TopA were minimal and unlikely to be of practical significance.

These results suggest that ADVI can provide sufficiently accurate posterior estimates for BMCA in both simple and moderately complex models. While HMC remains the gold standard for posterior accuracy, the consistency between ADVI and HMC in most cases supports the use of ADVI as a practical and computationally efficient alternative.

Based on this, we concluded that the combination of the v-based formulation and ADVI inference yields results comparable to those of previous BMCA implementations. We therefore adopted this framework to investigate how different types of input data influence the accuracy and robustness of BMCA predictions.

#### Posterior support for ground truth values.

To complement our evaluation of predictive accuracy using point estimates, we also computed the log probability density (logp) of the ground truth parameter values under the inferred posterior distributions for each perturbation dataset for TopA and TopB without allosteric regulators (Fig D in S1 Text). Specifically, posterior samples from the PyMC trace were used to approximate the marginal posterior density at each elasticity value via kernel density estimation, and the logs of these densities were recorded. Most ground truth elasticities had positive logp scores, indicating that the posterior distributions assigned reasonably high probability to the correct values. A smaller subset had negative logp values, reflecting lower confidence in those predictions. On the whole, the distribution of logp scores leans positive with a long negative skew.

### Impact of input data on BMCA predictions

#### Omitting different data types leads to different BMCA elasticity predictions.

Elasticity values generated by BMCA in various conditions and model variations were compared to ground truth to identify factors that influenced the BMCA algorithm’s performance. The mean of each HDI from each posterior distribution for each elasticity was calculated for comparison.

**Fig 4:**
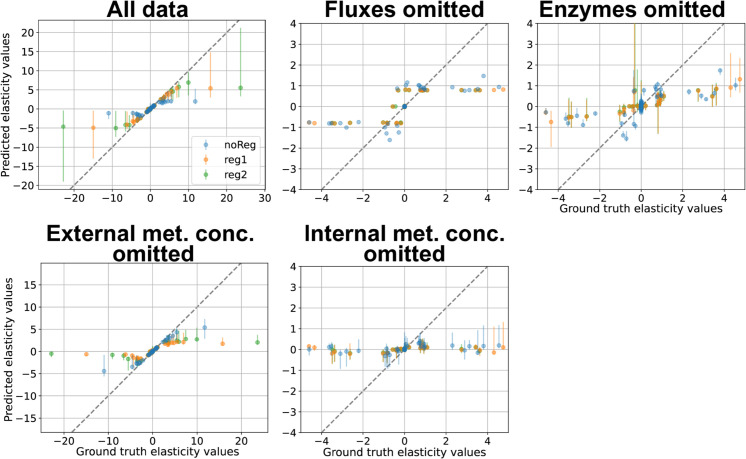
BMCA elasticity predictions compared against ground truth values for TopB. Each dot signifies the median while the error bars represent the range of the predicted elasticity values across the different enzyme perturbation levels tested. The titles for each graph indicate which data type was omitted when running BMCA. All of the graphs are zoomed in as the ground truth elasticity.

In TopA, when BMCA was given all the data, the predicted elasticity values matched the ground truth values well, even for variations with allosteric regulation (Fig E in S1 Text). The absence of internal metabolite concentrations led BMCA to predict nearly all elasticities as zero. When enzyme concentrations were omitted, elasticities with negative ground truth values were predicted close to zero, while those with positive ground truth values were underestimated. Conversely, omitting external metabolite data had minimal impact on the algorithm’s performance, as the results remained nearly identical to those obtained with all data types included. Additionally, despite not receiving flux data, the BMCA algorithm was still able to determine whether an elasticity is negative or positive; however, it resulted in the majority of predicted elasticities being either 1 or –1. However, the predictions were identical across all of the perturbation levels. The same patterns were also observed in TopB (Fig 4). However, in TopB, only the elasticities whose ground truth values were within –2 and 2 were predicted well when the BMCA algorithm was given all the data or when the external metabolite concentrations were withheld from the algorithm. The elasticities outside of that range were not predicted well.

To evaluate the influence of individual metabolites on BMCA inference accuracy, we performed a sensitivity analysis on Topologies A and B (no allosteric regulators). This involved running BMCA on a full dataset where the enzymes were serially perturbed to 150% of their original wild type concentrations. For each metabolite, we created a variation of the full dataset by omitting its concentration data and ran BMCA on this modified input. The resulting elasticity predictions were compared to those from the full dataset by computing the RMSE against ground truth values. The differences in RMSE are shown in Fig F in S1 Text. Each metabolite was classified as either an internal linear metabolite (only one consuming reaction), an internal branching metabolite (multiple consuming reactions), or an external metabolite (boundary species in the model). For TopA, there is a general cascade effect in influence flowing from the beginning towards the end of the network. For TopB, the pattern is less clear. Generally, we found that the internal branching metabolites are interspersed quite evenly among all the candidate metabolites and the external metabolites tend to have little influence. While this metabolite-wise sensitivity analysis provides useful insights, the results are limited to the specific topologies examined. Broader generalizability would require additional studies across a wider range of network structures. Nevertheless, this approach may be useful in guiding experimental prioritization of metabolite measurements in future BMCA applications.

#### Ground truth elasticities with magnitudes larger than 1.5 are often underestimated.

**Fig 5:**
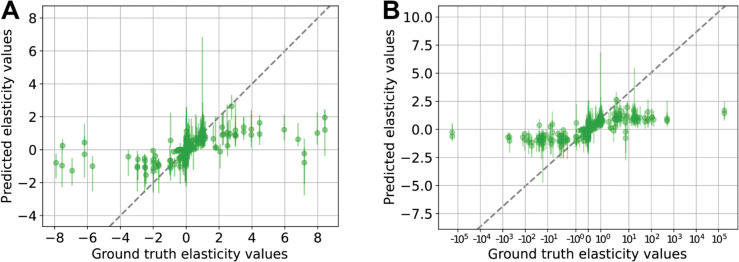
BMCA-predicted elasticities for TopC across all perturbation strengths compared against ground truth values. A) Comparison of elasticities for ground truth values less than a magnitude of 10. B) Comparison of elasticities for all ground truth values. Each dot signifies the median while the error bars represent the range of the predicted elasticity values across the different enzyme perturbation levels tested.

BMCA seems to demonstrate a range for which it can make good predictions for elasticity values. For all cases of providing the BMCA algorithm with all the data, the expectation is that the algorithm should predict the elasticity values correctly. This is observed in the elasticity prediction results for TopA when no data was omitted. However, providing complete data to the BMCA algorithm did not produce perfect elasticity predictions for TopB and C. The BMCA algorithm predicts well for a small range of values between –1.5 and 1.5. Since the ground truth elasticity values for TopA are small, the BMCA algorithm has no problem predicting all of its elasticity values. In contrast, Topologies B and C have much larger ground truth elasticity values and predictions for the ground truth elasticity values outside of the –1.5 and 1.5 range are capped at about –2 and 2 (Figs 4 and 5).

#### Effect of varying perturbation levels and regulator presence on elasticity predictions across network variations.

Levene’s test was used to determine whether elasticity predictions differed in variance across perturbation levels and varying degrees of allostery. This test assesses whether the groups being compared have equal variances. Variability in the data was analyzed instead of central tendency (ANOVA or Kruskal-Wallis test), as all sets of elasticity predictions, regardless of perturbation or allostery level, have a mean and median of zero.

Analysis of the BMCA results revealed that the algorithm’s elasticity predictions remained largely unaffected by variations in enzyme perturbation levels for TopA (Table 3 and Fig H in S1 Text). In contrast, for TopB variations with allosteric regulators, there were significant differences in the variance of elasticity predictions between different perturbation strength datasets, as indicated by Levene’s test. Increased perturbation strength to TopB enzymes resulted in better elasticity predictions (Fig H in S1 Text). For TopC, which contains numerous regulators, elasticity predictions varied significantly across all perturbation levels. However, when comparing elasticity predictions across different regulatory conditions (e.g. TopB-noReg, TopB-reg1, and TopB-reg2) at the same perturbation levels, no significant differences were observed.

**Table 3:** Results of Levene’s test comparing predicted elasticities across varying perturbation levels for different network variations. *n* is the Hill coefficient for the regulators. For Reg2 conditions, the two Hill coefficients are listed in order as Reg1 and Reg2.

Network	Condition	F-statistic	p-value
TopA	noReg	0.08	0.99
	Reg1 (n=1)	0.17	0.99
	Reg2 (n=1,1)	0.29	0.97
	Reg1 (n=4)	0.43	0.91
	Reg2 (n=4,3)	0.34	0.96
TopB	noReg	0.10	0.99
	Reg1 (n=1)	4.60	4.76e-6
	Reg2 (n=1,1)	4.23	1.86e-5
	Reg1 (n=3)	4.25	1.76e-5
	Reg2 (n=3,4)	5.31	3.30e-7
TopC	-	15.2	4.93e-25

#### Impact of allosteric regulator strength on BMCA-predicted elasticities.

Previous work [12] on BMCA demonstrated the potential for BMCA to uncover latent allosteric relationships, albeit with limited reliability. To further investigate BMCA’s ability to detect allosteric regulation, we used variations of TopA and TopB that incorporated one or two known allosteric regulators (reg1 and reg2 variants) without omitting any data. TopC was excluded from the Hill coefficient analysis due to the presence of multiple allosteric regulators, which made it difficult to isolate and interpret the individual effects of each regulator. Initially, the allosteric interactions were weak, with Hill coefficients set to 1. To strengthen regulatory effects, we increased the Hill coefficients in the network models used to generate the synthetic perturbation datasets (Table 4). These modifications introduced more nonlinear behavior into the ground truth by simulating stronger cooperativity prior to data generation. BMCA was then applied as before, using a lin-log model to infer elasticities from these perturbed datasets. This allowed us to assess BMCA’s ability to recover accurate elasticity estimates when the true system deviates from the lin-log assumption, particularly under stronger allosteric regulation.

**Table 4:** Ground truth elasticity values before and after allostery was increased for TopA and B. Ground truth elasticity values are provided for the allosteric regulators at different Hill coefficients (*n*).

Model variation	Allosteric elasticity	*n*	Ground truth value
TopA-reg1	$\varepsilon_{J}^{OSC}$	1	–0.680
		4	–3.611
TopA-reg2	$\varepsilon_{J}^{OSC}$	1	–0.667
		4	–3.517
	$\varepsilon_{G}^{LIM}$	1	–0.655
		3	–2.201
TopB-reg1	$\varepsilon_{H}^{v5}$	1	–0.566
		3	–2.604
TopB-reg2	$\varepsilon_{H}^{v5}$	1	–0.566
		3	–2.605
	$\varepsilon_{O}^{v14}$	1	–0.439
		4	–3.278

**Fig 6:**
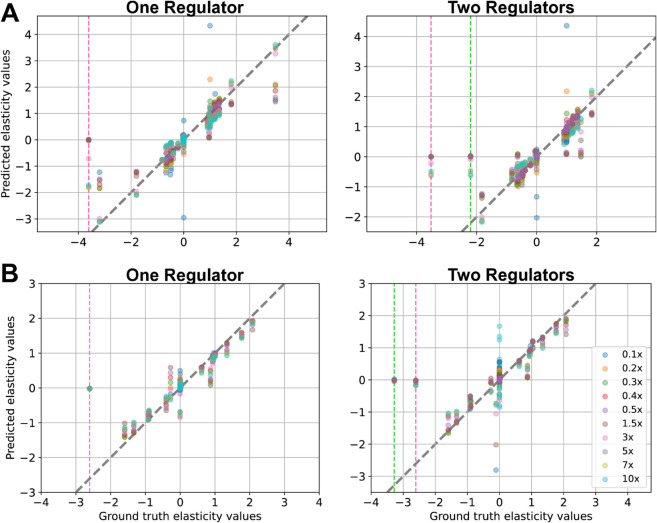
Elasticity predictions for model variations with strong allosteric regulators. BMCA-predicted elasticities from ten different perturbation strength datasets for (A) TopA and (B) TopB, each with one or two strong allosteric regulators (Hill coefficient = 3 or 4). Each dot represents the median of the predicted distribution means across the ten enzyme perturbation strengths. The pink dashed line indicates the ground truth elasticity of the first regulator, and the green dashed line indicates that of the second regulator.

BMCA failed to accurately predict the elasticities associated with allosteric regulators in both TopA and TopB, regardless of the number of regulators (Fig I in S1 Text). Even after strengthening the allosteric relationships by increasing Hill coefficients, the model continued to underestimate the true elasticity values, often predicting values near zero (Fig 6A). In contrast, the accuracy of elasticity predictions for non-regulatory metabolites, particularly those with ground truth values between ±2, improved in TopB as perturbation strength increased (Fig 6B). This trend was most apparent in TopB-reg1, though not consistently observed in other model variations.

To determine whether this failure to recover allosteric elasticities was specific to the v-based implementation, we also tested the original *emll* code provided by St. John et al. on the TopA model with a strong allosteric regulator. Across ten perturbation levels, *emll* also failed to consistently identify the regulatory interaction (Fig 3D). While nonzero predictions were occasionally observed, the majority of runs yielded elasticity estimates near zero, mirroring the performance of the v-based approach.

Ultimately, both implementations of BMCA failed to recover strong allosteric effects, even under idealized, noise-free conditions.

#### Comparison of BMCA CCC predictions with ground truth values.

**Fig 7:**
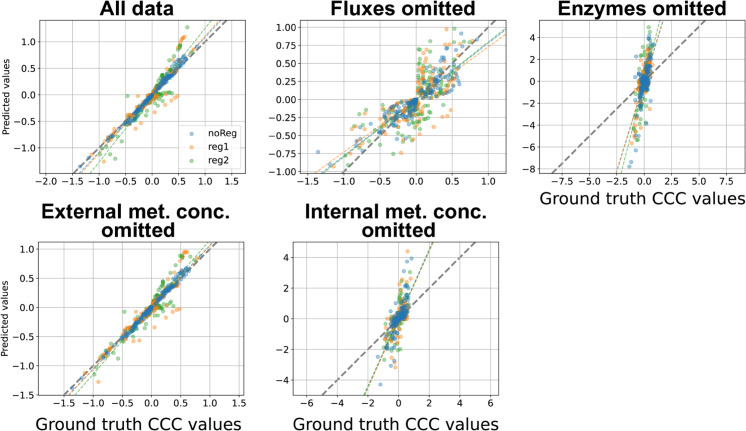
BMCA CCC predictions compared against ground truth values for TopA. Each dot signifies the median of the predicted CCC values for the different enzyme perturbation levels tested. The titles for each graph indicate which data type was omitted when running BMCA.

The BMCA algorithm’s predictions of CCCs for TopA aligned with its elasticity predictions in several ways (Fig 7). When provided with all data, predictions were accurate. Adding allosteric regulation reduced precision, while omitting fluxes generally preserved the correct sign of ground truth values but produced inconsistent and uninformative results otherwise. Omitting enzymes and internal metabolites caused overestimation, particularly for ground truth CCCs near zero. Excluding external metabolites had minimal impact, yielding results similar to using the full dataset.

For TopB, similar trends emerged but on a larger scale (Fig J in S1 Text). As with TopA, when no data was omitted, predictions matched ground truth values closely, particularly in datasets without regulators. Also, omitting fluxes preserved the sign of ground truth values but resulted in poor predictions while omitting enzymes and internal metabolites led to significant overestimation of CCCs. Excluding external metabolites did not degrade the BMCA algorithm’s predictive accuracy, even with the datasets that demonstrated implicit allosteric regulation.

Although the BMCA algorithm was provided with the complete dataset for TopC, it produced numerous nonzero CCC values where the ground truth values were zero, and vice versa (Fig K1 in S1 Text). The ground truth CCC values ranged from just above 2 to just below –2, with the predictions remaining within these bounds. However, the uncertainty ranges were large, sometimes exceeding ten times the predicted value (Fig L2 in S1 Text), rendering them uninformative.

#### Comparison of FCC predictions with ground truth values.

The omission of various data types significantly impacted the FCC predictions made by the BMCA algorithm. In TopA, no discernible difference was observed between providing all data and omitting external metabolite concentration data, suggesting that the addition of external metabolite concentration data only provide a modest impact to BMCA FCC predictions at this scale (Fig L in S1 Text). Similarly, omitting internal metabolite concentrations resulted in slightly more variance in the FCC predictions, although the overall effect was minimal. The exclusion of enzyme data resulted in some instances of FCC overestimation, although the majority of the data points were predicted within a 0.1 margin of their respective ground truth values.

The uncertainty ranges for the FCC predictions were uninformative and varied wildly when there were no data omissions or when enzyme data or internal metabolite concentration data was withheld from the BMCA algorithm (Fig M in S1 Text). When the flux data was omitted, the resulting uncertainty ranges did not cover the actual ground truth FCC values. Omitting only external metabolite concentrations led to reasonable uncertainty ranges that included the ground truth FCC values.

**Fig 8:**
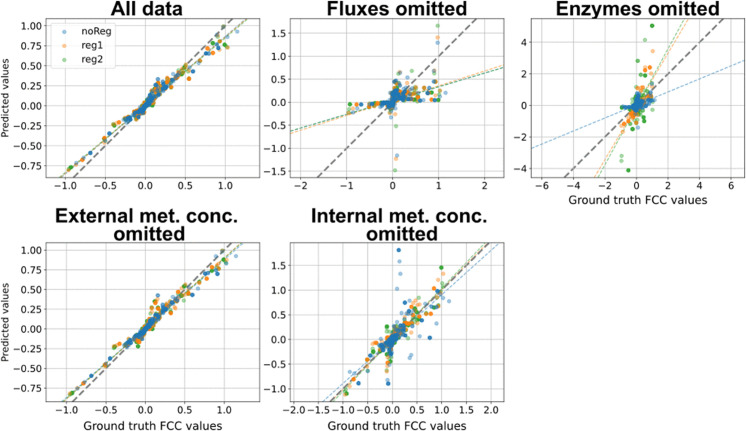
BMCA FCC predictions compared against ground truth values for TopB. Each dot signifies the median across the different enzyme perturbation levels tested. The titles for each graph indicate which data type was omitted when running BMCA. Corrections for FCC values for reactions whose enzymes are being perturbed have been applied.

For TopB, which features longer network branches than TopA, the FCC prediction behaviors observed in the omission experiments were similar in pattern but exhibited larger magnitudes (Fig 8). Supplying the BMCA algorithm with all available data in the absence of allosteric regulation resulted in posterior predictions close to their respective ground truth values. However, model variations with allosteric regulation exhibited some overestimation in FCC values.

Excluding the flux data had a more pronounced impact, leading to decreased precision in the predicted FCC values, particularly as the ground truth values deviated further from zero. BMCA both underestimated and overestimated the magnitude of FCC values under these conditions, and the omission of flux data yielded little to no change between the prior and posterior distributions for both TopA and B (Fig N in S1 Text). These results indicate that flux data is essential for accurate FCC predictions.

While acceptable predictions were made in TopA, possibly due to the smaller ground truth elasticity and FCC values in the network, excluding fluxes in TopB led to BMCA predicting seemingly random values that roughly matched the sign (positive or negative) of ground truth values. These predictions were neither accurate nor informative, further reinforcing the conclusion that flux data is essential for accurate FCC predictions.

In contrast, excluding internal metabolite concentration data in TopB led to FCC predictions that were close to their respective ground truth values across all allosteric variations (Fig 8). While the elasticities predicted for model variations with more allosteric regulators exhibited more variance, nearly all predictions were close (within 0.5) of their ground truth values. This finding aligns with the observation in TopA, supporting the conclusion that BMCA does necessarily need internal metabolite concentrations to make FCC predictions given all other types of data. Excluding enzyme data in TopB resulted in many overestimated FCC predictions, a pattern that paralleled the underestimation of the elasticity values when the enzyme data was omitted. This underscores the importance of enzyme data in obtaining good predictions for both elasticities and FCC values.

Finally, the FCCs predicted by BMCA for TopC showed improved accuracy compared to those based on prior distributions, particularly by reducing the overestimation of values near zero. (Fig O1 in S1 Text). The median FCC values across different perturbation strengths fell within the corresponding uncertainty ranges; however, the uncertainty ranges remained too large to provide meaningful insights (Fig O2 in S1 Text).

#### Evaluating FCC rankings predicted by BMCA.

**Fig 9:**
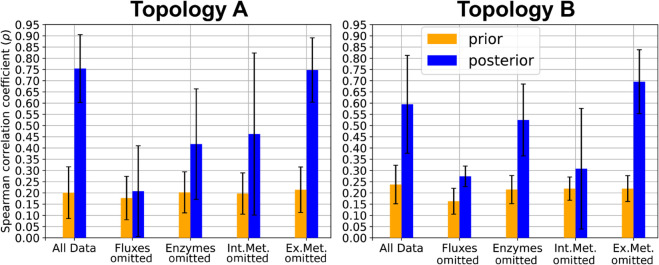
Aggregated Spearman correlation coefficients from comparisons between ground truth rankings of FCC values and FCC values predicted by the BMCA algorithm.

The informativeness of a single FCC value is limited. To determine the significance of a reaction’s FCC value, it must be compared to the FCCs of all other reactions in the network. Given the practical constraints of flux and enzyme concentration data availability, this study aimed to evaluate whether the predicted FCC values maintained their relative magnitudes in accordance with the order of ground truth FCC values. To assess the influence of different data types on the BMCA algorithm’s ability to predict FCC values and rankings, Spearman correlations were calculated between predicted and ground truth rankings across runs with varying allosteric regulators and omitted data types (Fig 9).

**Fig 10:**
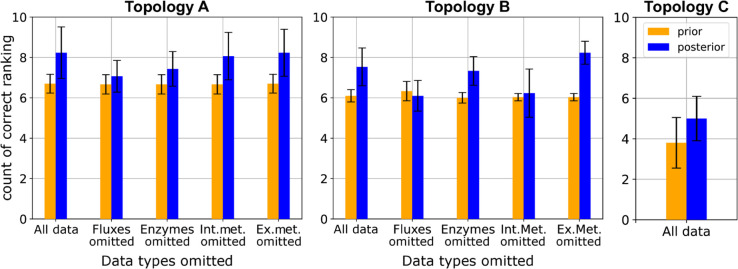
Number of enzymes correctly predicted as having one of the top ten highest FCC values based on the various data types omitted when running BMCA. The height of the bars represent the mean of the predictions across all the perturbation levels and allosteric regulation levels; the error bars represent the standard deviation.

The difference in the change of correlations between the prior and posterior distributions were significant for many of the different data-type input trials, suggesting that the BMCA algorithm changed the relative FCC values based on the inputted data. The highest FCC values are the most useful for engineering strains, as reactions with these values represent the most promising targets. Therefore, the ten reactions with the highest predicted FCC values were examined to determine if they aligned with the order of the ground truth FCC values. Across all of the different variations of data-type inputs to the BMCA algorithm and the perturbation levels of the inputted data, the average number of correctly predicted reactions predicted to be in the top ten ranged between 5 and 9.4 (Table A in S1 Text). Notably, omission of flux data produced identical overlap values across perturbation strengths (SD = 0), while the exclusion of external metabolite data consistently resulted in a higher average than when given all the data. Omission of other data types yielded more variable outcomes. There was also little difference between the number of reactions that were predicted to be in the top ten calculated from the prior and posterior distributions. Only when all data was made available or the external metabolites were omitted was there a definitive improvement in FCC ranking prediction (Fig 10). While the omission of enzyme concentration and internal metabolite concentration data resulted in minor improvements in predicting the top ten FCC enzymes, there was greater statistical overlap with prior counts. Notably, there was no change in identifying the top ten ranked FCCs when flux values were omitted between the prior and posterior predictions. This pattern persisted even in the presence of allosteric regulation (Fig P in S1 Text).

Similarly, for TopC, there was a marginal increase between using the prior or posterior distribution to calculate the number of correctly identified reactions with the highest FCCs when all data types were provided to BMCA. BMCA was able to predict at least 4 of the top 10 reactions with the highest FCC rankings for TopC, and inputting all possible data into the BMCA algorithm only improved the prediction of the top ten FCCs by one more metabolite.

Analysis of all FCC values predicted by BMCA for TopC reveals that the ranking of FCC values is predominantly influenced by the observed reaction rather than the perturbed enzyme. This observation is supported by the distinct vertical patterns evident in the heatmap (Fig Q in S1 Text). These patterns suggest that the underlying network structure exerts a significant influence on FCC values, potentially overshadowing the direct effects of individual enzyme perturbations.

The true rankings of the enzymes that the BMCA algorithm incorrectly identified as being in the top ten were further investigated across the ten different perturbation levels tested on TopC without any omissions of any data (Fig R in S1 Text). The BMCA algorithm most frequently predicted that “XCH_ACE1,” the reaction transporting acetate out of the cell into the periplasm, and “XCH_P,” the reaction transporting phosphate into the cell from the periplasm, were in the top ten. However, these reactions’ ground truth FCC rankings were 57 and 61st, respectively.

## Discussion

Our study aimed to evaluate the predictive capabilities and limitations of BMCA in determining key control points within metabolic pathways, particularly in data-limited scenarios. By systematically assessing the impact of different physiological data types on the BMCA algorithm’s accuracy at predicting control coefficients and allosteric interactions with our three synthetic models, we provide a clearer understanding of its strengths and constraints. Although BMCA’s posterior distributions were sometimes broad or truncated, the logp analysis (Fig D in S1 Text) shows that the method nonetheless assigns nontrivial probability to many true values, providing a distribution-level validation of the inference process. Overall, given sufficient data, we demonstrate that BMCA can make reasonable predictions of control coefficient values and relative orders, particularly for systems whose true elasticity values do not exceed 1.5, when flux and enzyme concentration data are available in abundance, and when allosteric regulation is limited or absent (Fig 4). We did not find any instances in which BMCA correctly inferred the elasticity of an allosteric regulator (Fig 6). And while the rankings of the FCC values improved after applying BMCA, this appeared to reflect its ability to partially recover some of the correct values, rather than capturing a systematically scaled version of the ground truth (Fig 9).

This work contributes to the broader effort of optimizing metabolic engineering by offering guidance on when and how BMCA can be effectively applied. More generally, our findings underscore the potential of mechanistic models to provide biological insight while requiring less extensive data collection compared to machine learning approaches, positioning them as valuable tools for accelerating the rational design of biomanufacturing processes.

### Impact of data availability on BMCA predictive accuracy

An important consideration in the application of BMCA is its robustness to missing data, given the challenges associated with obtaining experimental measurements. Understanding the relative importance of different data types can provide valuable guidance for prioritizing data collection efforts. The impact of omitting specific data types was assessed across different network topologies, revealing distinct effects on model accuracy (Table 5).

**Table 5:** Summary of effects on different control coefficients when different types of data types are omitted from BMCA.

Data Omitted	Elasticities	CCCs	FCCs
**None**	Predictive	Predictive	Predictive, with some poor estimation
**Fluxes**	Signs only	Uninformative	Uninformative
**Enzyme Concentrations**	Underestimation	Poor estimation	Poor estimation
**Internal Metabolites**	Underestimation	Poor estimation	Predictive
**External Metabolites**	Predictive	Predictive	Predictive

Omitting external metabolite data had little effect on the predictive accuracy of the BMCA algorithm for both TopA and B across all metrics.

The omission of enzyme data had a more pronounced effect. Elasticity predictions were underestimated, often nearing zero, suggesting that enzyme values provide critical scaling information. Without enzyme data, both CCC and FCC values were poorly estimated. Unlike internal metabolite data, the lack of enzyme information appears to create a scaling issue that persists through the FCC calculations, as the scaling factor was missing in the elasticity-to-CCC transformation.

Flux data was crucial for accurate predictions across all metrics in both topologies. Excluding flux data degraded the quality of elasticity, CCC, and FCC predictions, preserving only the sign of the ground truth values while failing to capture their magnitudes. The elasticity and FCC predictions also demonstrated the crucial role flux data plays in informing the BMCA algorithm’s predictions. There was no difference between the prior and posterior distributions without the flux data, even with the presence of all other types of data (Fig N in S1 Text). Flux data, like enzyme data, plays a key role in scaling elasticities during CCC calculations. Its absence leads to compounded inaccuracies, making FCC predictions particularly unreliable.

Internal metabolite data played a significant role in elasticity and CCC predictions. Without this data, elasticity values were strongly underestimated, often near zero, reflecting a loss of identifiability for these parameters. Since CCCs are derived from elasticities, they were subsequently poorly estimated. Interestingly, FCC predictions remained accurate despite the omission of internal metabolite data. This outcome suggests that while individual elasticities can become less identifiable without internal metabolite data, the ratios between the elasticities which are used to compute FCCs remain structurally identifiable under the available data (fluxes, and enzyme concentrations, and external metabolite concentrations).

This structural identifiability of the lin-log model could be a limitation of BMCA if using BMCA to predict elasticities or CCCs. In the lin-log framework, steady-state fluxes are expressed as linear combinations of log-transformed metabolite concentrations, each scaled by an unknown elasticity coefficient. When multiple elasticities contribute to a single flux equation, the system becomes underdetermined, meaning that multiple sets of elasticity values can yield identical flux predictions. This is a structural issue and persists even with perfect data. These findings underscore the challenge of recovering unique parameter estimates for elasticities, particularly when input data are sparse or only weakly informative.

This conclusion is further supported by (Figs 3A and 3C), where elasticity values obtained from both the *emll* and the v-based methods differed substantially, yet the FCC predictions remained nearly identical. Notably, the *emll* approach uses metabolite concentrations close to the unperturbed reference state rather than those from the perturbed state. St. John et al. [12] noted that this choice aids in the parameter identifiability of FCCs. We hypothesize that it may also improve identifiability by reducing the propagation of noise through the calculations, particularly when working with experimental data that inherently contains measurement variability. By anchoring calculations to consistent metabolite concentrations, *emll* likely stabilizes inference and mitigates the compounding effect of noise, even if the individual elasticity parameters do not perfectly characterize the system.

In conclusion, external metabolite data is largely non-critical for BMCA predictions and may even be detrimental in some systems. In contrast, flux and enzyme data are essential for accurate predictions, with their omission causing significant errors. Internal metabolite data plays a crucial role in correctly estimating elasticities and concentration control coefficients, but its absence does not appear to strongly affect the identifiability of FCCs. Finally, network complexity amplifies the effects of data omissions, especially for flux and enzyme data, as observed in TopB.

### Elasticity limitations

Understanding metabolic network behavior requires accurate predictions of elasticity values, which describe how reaction rates respond to changes in metabolite concentrations. The BMCA algorithm was developed to estimate these values, but its accuracy varies depending on reaction topology and elasticity range. While it performs well for reactions with small elasticity values, it exhibits reduced accuracy with larger elasticities, particularly outside the range of –1.5 to 1.5. In these cases, the algorithm systematically underestimates values, capping predictions around ±2 (Fig 4). This limitation is especially pronounced for reactions near equilibrium, where elasticity magnitudes of 10 or greater are often reduced to 1.5 or lower (Fig 5). The comparison between HMC and ADVI predictions suggest that the elasticity cap at 1.5 is an artifact of ADVI (Figs C1 and C2 in S1 Text); HMC predictions stray less from the ground truth at higher magnitude elasticity values, though the prediction accuracy does increase with stronger perturbation strengths, indicating that both inference method and perturbation strength can jointly affect performance. These findings suggest that BMCA is more reliable for moderate elasticity ranges but requires improvement in capturing extreme values.

This pattern may arise because even substantial enzyme perturbations can lead to only modest shifts in metabolite concentrations and fluxes. Such muted responses may be due to buffering mechanisms, including reactions operating near equilibrium, which limit the observable dynamic range of the system. Since BMCA estimates elasticities by fitting a linear relationship between relative changes in flux and the natural log of relative changes in metabolite concentrations, this constrained variability in both quantities may restrict the slope that can be inferred. Although a truly high elasticity would imply that small changes in metabolite concentration produce large changes in flux, saturation effects may flatten this relationship in practice. The apparent cap in inferred elasticity values may also depend on the network structure: for example, in our simulations, TopB exhibited an upper bound around ±2, while TopC exhibited a tighter range around ±1.5. As a result, BMCA may systematically underestimate elasticities in systems where the true sensitivities are high but the observed system-level responses are buffered or limited.

To enhance BMCA’s predictive utility, it is essential to determine an appropriate range for elasticity values. One way to do this is by analyzing the disequilibrium ratio (*ρ*), which quantifies how far a reaction is from equilibrium. Since reactions near equilibrium contribute minimally to metabolic control, accurately predicting their elasticity coefficients is less critical. Instead, it is more important for the BMCA algorithm to prioritize identifying reactions further from equilibrium, which are more relevant for strain design. The disequilibrium ratio is defined as the mass-action ratio divided by the equilibrium constant (Eqs 3, 4) [23], where the product and substrate concentrations determine the deviation from equilibrium. When ρ=1, the reaction is at equilibrium. When ρ<1, the reaction is out of equilibrium in the forward direction. Likewise, when ρ>1, the reaction is out of equilibrium in the reverse direction. The magnitude of elasticity values depends on the disequilibrium value (Eq 5), where *p* represents the concentration of the *i*-th product, and *s* represents the concentration of the *i*-th substrate. Large-magnitude elasticities typically correspond to *ρ* values close to 1, signifying near-equilibrium conditions, while small-magnitude elasticities indicate reactions further from equilibrium (17).

$$\Gamma =\frac{p_{1}p_{2}...}{s_{1}s_{2}...},$$ (3)

$$\rho =\frac{\Gamma }{K_{eq}},$$ (4)

$$\varepsilon_{s}^{v}=\frac{1}{1-\rho }$$ (5)

BMCA’s current elasticity predictions, where the magnitudes of the elasticities are capped at 2, constrain the predicted disequilibrium ratio to between 0.5 and 1.5 in the forward and reverse direction, respectively. However, reactions with elasticity magnitudes greater than 2 can still be far from equilibrium, meaning that this cap leads to underestimated elasticities and, consequently, overestimated CCCs and FCCs.

### Predicting allosteric regulators

While the ability to infer allosteric regulation from latent data patterns represents a promising feature of BMCA, the algorithm consistently failed to detect allosteric interactions across multiple synthetic models, even in cases where the regulatory effects were pronounced. These findings raise important questions regarding the specific conditions under which BMCA can successfully infer allosteric regulation, particularly in light of previous studies [12] that either detected only a single allosteric interaction in a medium-sized network or required a highly simplified system consisting of only three reactions and three metabolites. To assess whether the inability to recover strong allostery was specific to the inference engine, we ran HMC on TopB with a single regulator (n = 3). As shown in Fig G in S1 Text, both ADVI and HMC yielded nearly identical posterior elasticity estimates, neither recovering the ground-truth allosteric elasticity. Together with the observation that both v-based and *emll*-based BMCA implementations fail under noise-free conditions, this result suggests that the limitation arises from model structure and identifiability (e.g., the lin-log approximation and data informativeness) rather than the inference algorithm.

Additionally, the presence of a single high-value allosteric elasticity in the system appeared to allow BMCA to overcome its typical constraint of predicting elasticity values within the range of –1.5 to 1.5, suggesting that larger perturbations may enhance predictive accuracy. This outcome is unexpected because, despite the presence of several non-allosteric elasticities with higher magnitudes in TopC, their predicted values remain constrained within the 1.5 magnitude limit. Although predictions remained inaccurate even when allosteric regulation strength was high, the overall accuracy of elasticity value predictions improved, indicating that BMCA extracts meaningful relationships between metabolites from the data but is unable to explicitly attribute these relationships to specific allosteric interactions.

### Effect of perturbation and allostery levels on BMCA predictions

Perturbation levels appear to have little effect on TopA, possibly due to its smaller elasticities, which may facilitate accurate predictions even in the presence of allosteric regulators. In contrast, for TopB and TopC, perturbation levels influence elasticity predictions, particularly when allostery is introduced. The extent of allostery does not appear to affect the predictive performance of the BMCA algorithm; rather, perturbation strength plays a more significant role. The finding that stronger perturbations enhanced prediction accuracy (Fig H in S1 Text) was also echoed in Ito et al.’s work [24]: the authors observed better predictive performance with larger perturbations on their machine learning framework that relied on local linear approximations to make predictions. Further investigation is necessary in determining which perturbation levels yield more accurate predictions and understanding the underlying reasons.

### Impact of network structure on FCC ranking fidelity

While absolute FCC values can provide useful insights when considered in the context of the entire system, an alternative approach is to rank all FCC values by magnitude to identify the most influential reactions. To evaluate the ranking fidelity of the BMCA algorithm, its ability to correctly identify the top-ranking FCC values was assessed. The results indicated that providing additional data led to only limited improvements in the accuracy of the top ten FCC predictions. Furthermore, predicted FCC rankings remained largely unchanged regardless of which enzyme was perturbed during data collection. These findings suggest that network structure plays a fundamental role in determining FCC values within BMCA, highlighting the need for further investigation into its influence on metabolic control predictions.

### Future work

At present, BMCA’s ability to estimate metabolic control analysis coefficients remains limited. To extend this work, future research could more extensively explore added noise in the data tested, employ different prior distributions, and investigate the combination of flux and internal metabolite concentrations necessary for BMCA to provide a more comprehensive understanding of the limitations of BMCA.

The influence of prior distributions remains an open question, as current results exhibit a strong bias toward predicting zero for all elasticities and a tendency to constrain estimates within the range of –1.5 to 1.5. Testing alternative prior distributions that would expand the BMCA algorithm’s predictive range for elasticities from –5 to 5 could improve its estimation accuracy and better capture the elasticity values whose substrate disequilibrium ratios fall within 1± 0.2. Alternatively, since the v-based and *emll* methods exhibit differing ranges of elasticity prediction fidelity, a combined approach may leverage their complementary strengths to improve accuracy across a broader range of elasticity values.

In addition to addressing the elasticity cap, future improvements to BMCA could involve incorporating structural priors or additional modeling constraints to mitigate identifiability issues that arise in complex metabolic networks.

To further assess BMCA’s practical applicability, introducing controlled levels of noise into the ground truth data could help evaluate the method’s robustness under realistic experimental conditions, where measurement variability is inevitable. BMCA currently requires steady-state data, verified by the multiplication of the stoichiometric matrix (*N*) with the reference fluxes (v^*^), but assessing its sensitivity to deviations from steady-state assumptions could further refine its applicability.

BMCA’s inability to detect allosteric regulation remains a key limitation. Future work should aim to identify the specific conditions under which BMCA can successfully infer allosteric interactions. Investigating whether strong perturbations or particular network configurations influence allosteric detection could provide insight into the algorithm’s constraints.

By addressing these areas, future research can refine BMCA’s predictive capabilities, improve its ability to infer metabolic control mechanisms, and clarify the conditions under which it is most effective.

## Supporting information

### Supplementary figure legends

**FigA in S1 Text. BMCA predictions of elasticity coefficients, CCCs, and FCCs for Topology B with and without external metabolite perturbations.**

**FigB in S1 Text. Difference in corrected and uncorrected FCC predictions.**

**FigC in S1 Text. Comparison of HMC and ADVI elasticity predictions and variances.**

**FigD in S1 Text. Distributions of log probability density for different perturbation strength datasets.**

**FigE in S1 Text. BMCA elasticity predictions compared against ground truth values for Topology A.**

**FigF in S1 Text. Change in RMSE between BMCA elasticity predictions and ground truth when individual metabolites are omitted from the input data, relative to using all metabolite data.**

**FigG in S1 Text. Comparison of ADVI and HMC for Topology B with one allosteric regulator (n = 3).**

**FigH in S1 Text. Influence of perturbation strength on elasticity predictions.**

**FigI in S1 Text. Elasticity predictions for model variations with weak allosteric regulators.**

**FigJ in S1 Text. BMCA CCC predictions compared against ground truth values for Topology B.**

**FigK in S1 Text. CCC value comparisons for Topology C.**

**FigL in S1 Text. BMCA FCC predictions compared against ground truth values for Topology A.**

**FigM in S1 Text. Uncertainty ranges for BMCA FCC predictions compared against ground truth values for Topology A.**

**FigN in S1 Text. BMCA FCC predictions compared against ground truth values for Topology A and B model variations when flux values were omitted.**

**FigO in S1 Text. Topology C FCC value comparisons.**

**FigP in S1 Text. Number of enzymes correctly predicted as having one of the top ten highest FCC values based on the various data types omitted when running BMCA across different amounts of regulators.**

**FigQ in S1 Text. Accuracy of FCC rankings predicted by the BMCA algorithm for Topology C.**

**FigR in S1 Text. Ground truth rankings of enzymes that were incorrectly predicted as having a top ten highest FCC value for Topology C.**

### Supplementary table legends

Table A in S1 Text Top-10 overlap between predicted and ground truth FCC rankings. Values are reported as mean ± SD across replicates for each omission condition and topology.
(PDF)
