# Supplementary material for: Evaluating the limitations of Bayesian metabolic control analysis
Source: PLoS Comput Biol. 2026 Jan 16;22(1):e1012987. doi: 10.1371/journal.pcbi.1012987 (PMC12844504; doi:10.1371/journal.pcbi.1012987)
Supplement: Table A in S1 Text — Values are reported as mean ± SD across replicates for each omission condition and topology. (PDF) [file pcbi.1012987.s001.pdf]

Supplementary Information for:

Evaluating the limitations of Bayesian metabolic control analysis

Authors:

Janis Shin, James M. Carothers, Herbert M. Sauro

Correspondence:

hsauro@uw.edu

Code and Data Availability:

All code and datasets used in this study are available at https://github.com/sys-bio/BMCA-pipeline, version 2.1.0.

Figures

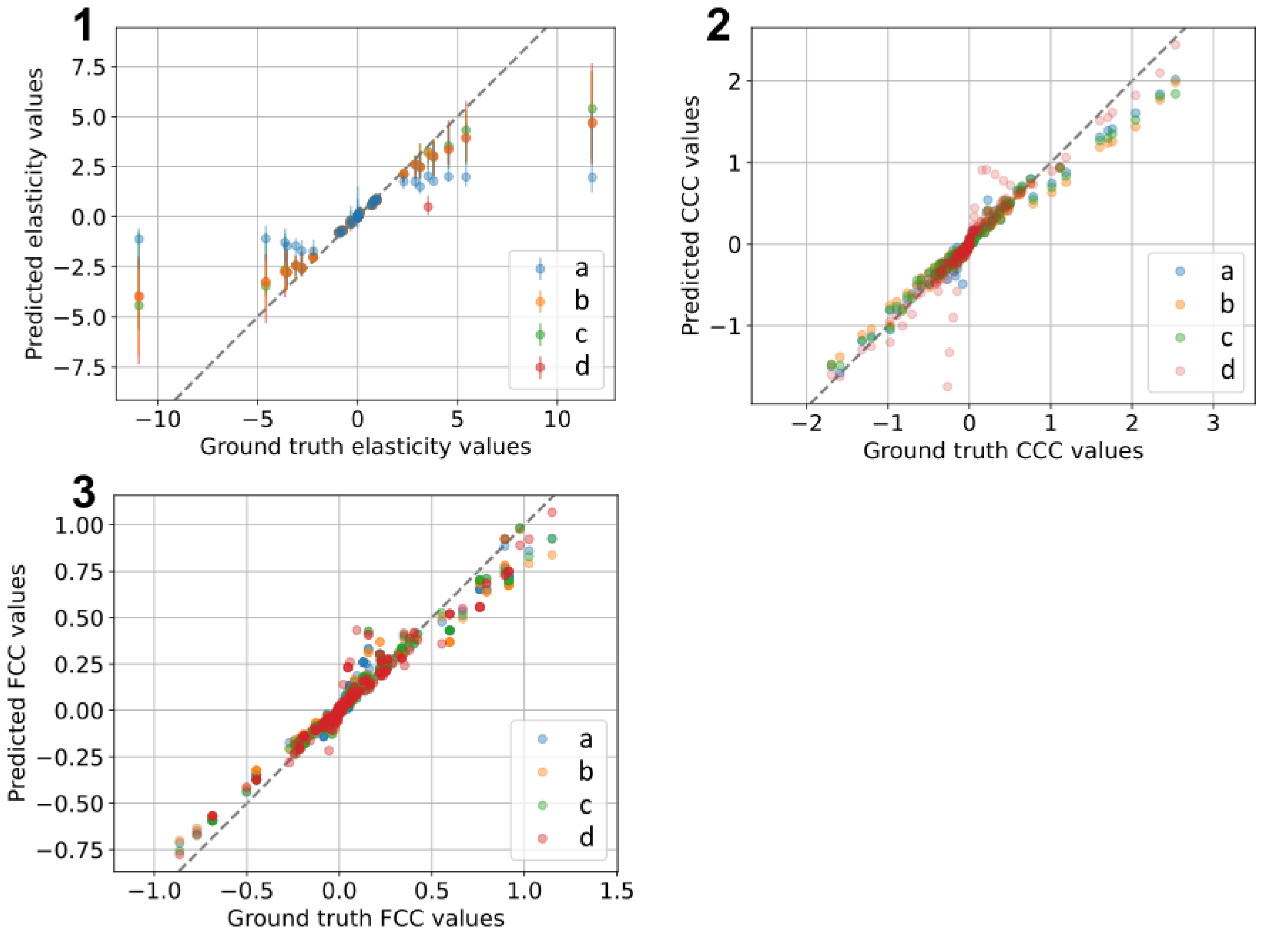

(Fig A) BMCA predictions of elasticity coefficients, CCCs, and FCCs for Topology B with and without external metabolite perturbations. ADVI was run across 10 different perturbation levels under four experimental conditions: a) Enzyme concentration perturbations only; b) Enzyme and external metabolite perturbations; c) Enzyme perturbations only, with external metabolite concentrations omitted from BMCA input; d) Enzyme and external metabolite perturbations, but with external metabolite concentrations omitted from BMCA input. 1) Median elasticity predictions are shown for each individual elasticity across the 10 datasets per condition, with error bars representing the full range. 2) Median CCC predictions per condition. 3) Median FCC predictions per condition. In all panels, the gray dotted line indicates perfect agreement between predicted and ground truth values.

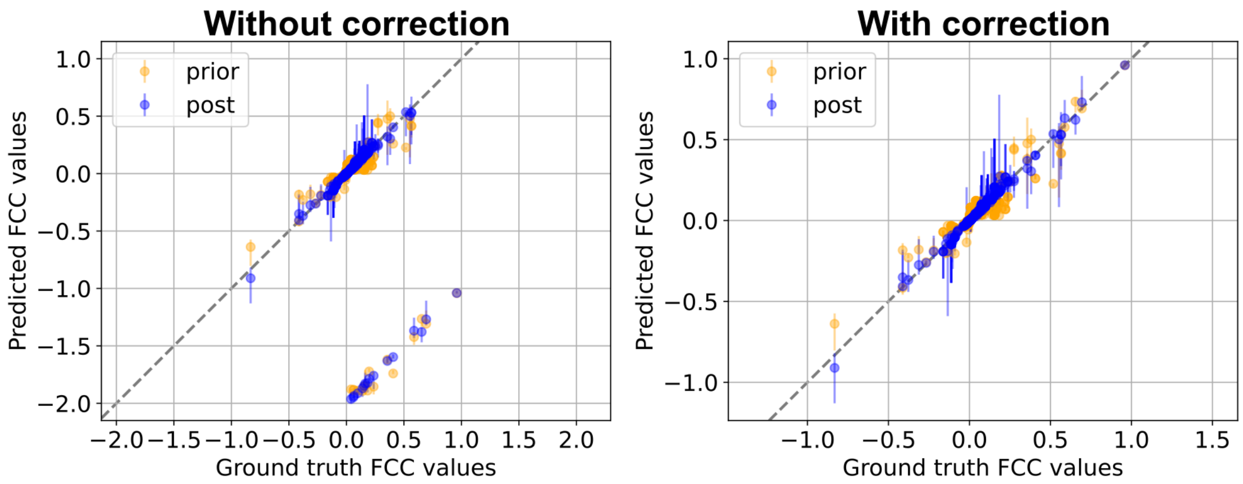

(Fig B) Difference in corrected and uncorrected FCC prediction. FCC predictions for Topology A with no allosteric regulators and no data withheld from BMCA with and without correction. Each dot signifies the median of the predicted values while the error bars represent the range across the different enzyme perturbation levels tested. The gray dashed line represents where the ground truth values match the predicted values.

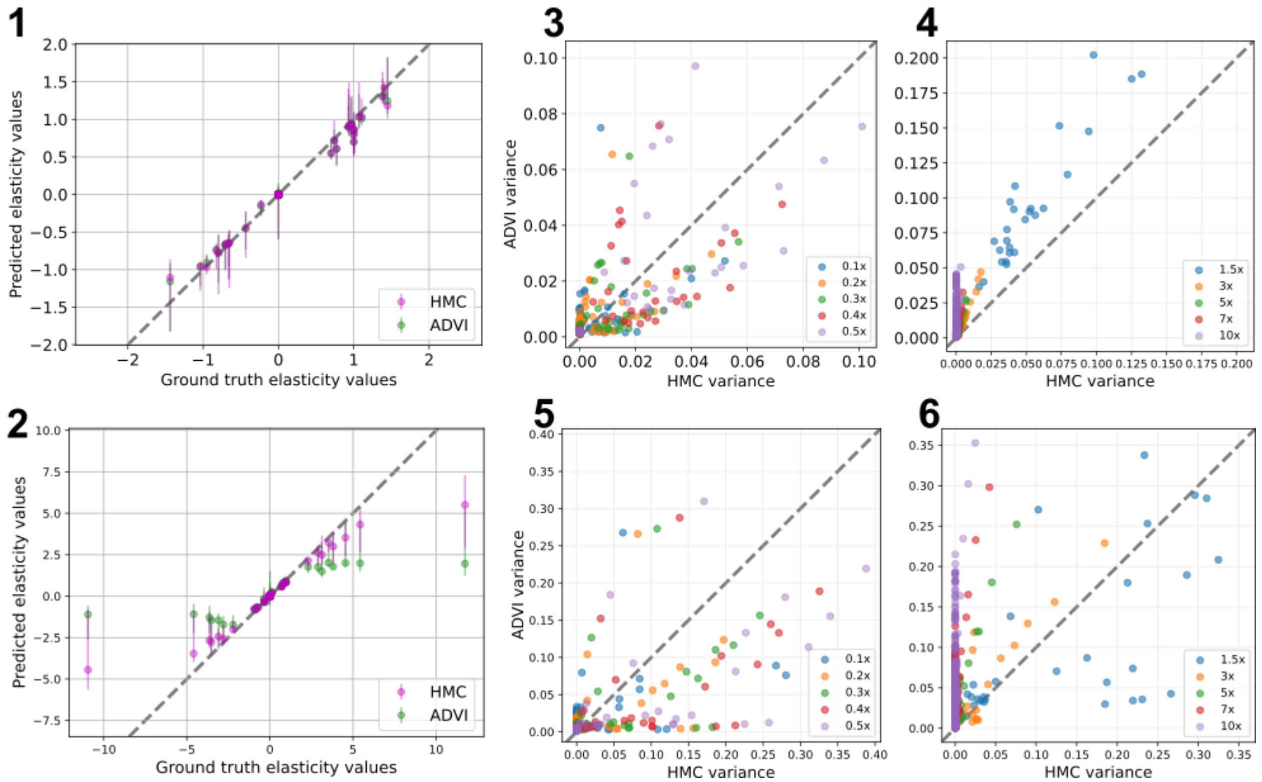

(Fig C) Comparison of HMC and ADVI elasticity predictions and variances. Elasticity predictions for (1) TopA-noReg and (2) TopB-noReg are shown. Dots represent the median prediction across different perturbation datasets, and error bars denote the range. The gray diagonal line indicates where predicted values match the ground truth. Variances of elasticity predictions are shown for HMC and ADVI under: (3) TopA-noReg knockdown perturbations, (4) TopA-noReg upregulation perturbations, (5) TopB-noReg knockdown perturbations, and (6) TopB-noReg upregulation perturbations. In these panels, each dot represents the variance of a predicted elasticity. The gray diagonal line indicates where HMC and ADVI variances are equal.

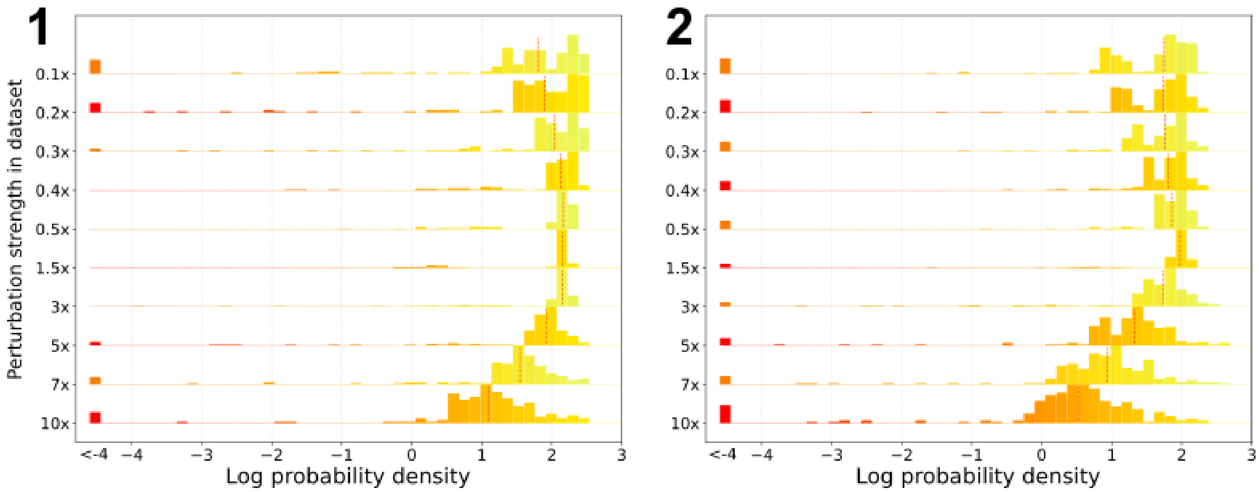

(Fig D) Distributions of log probability density for different perturbation strength datasets. (1) TopA-noReg and (2) TopB-noReg. Each histogram shows the distribution of logp values calculated for all elasticity predictions under a given perturbation strength. The red dashed vertical line indicates the median logp for that dataset. Logp values less than –4 are grouped into a single bin labeled "<–4".

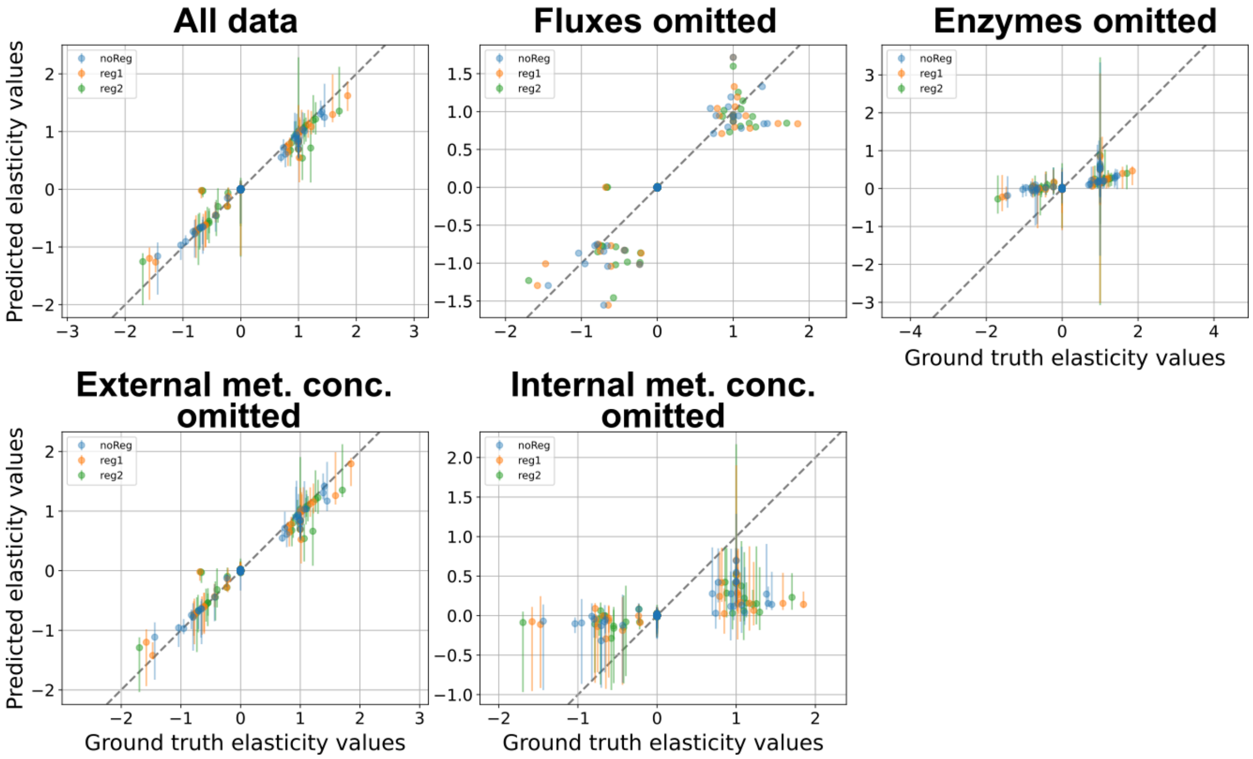

(Fig E) BMCA elasticity predictions compared against ground truth values for Topology A. Each dot signifies the median while the error bars represent the range of the predicted elasticity values across the different enzyme perturbation levels tested. The titles for each graph indicate which data type was omitted when running BMCA

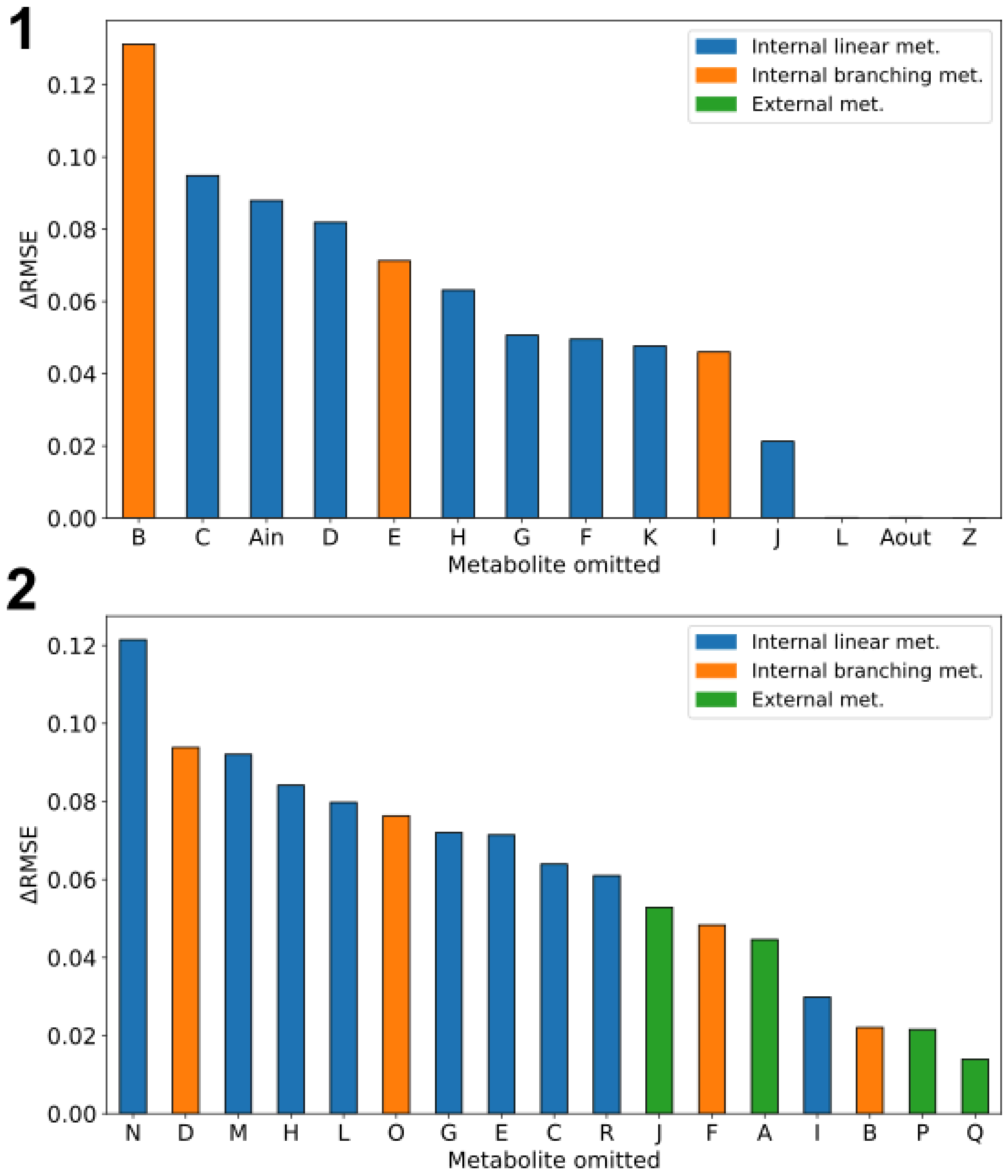

(Fig F) Change in RMSE between BMCA elasticity predictions and ground truth when individual metabolites are omitted from the input data, relative to using all metabolite data. (1) TopA-noReg; (2) TopB-noReg. The zero baseline indicates the RMSE obtained when all metabolite concentrations are included. Each metabolite is categorized as an internal linear metabolite (one consuming reaction), an internal branching metabolite (multiple consuming reactions), or an external metabolite (boundary species).

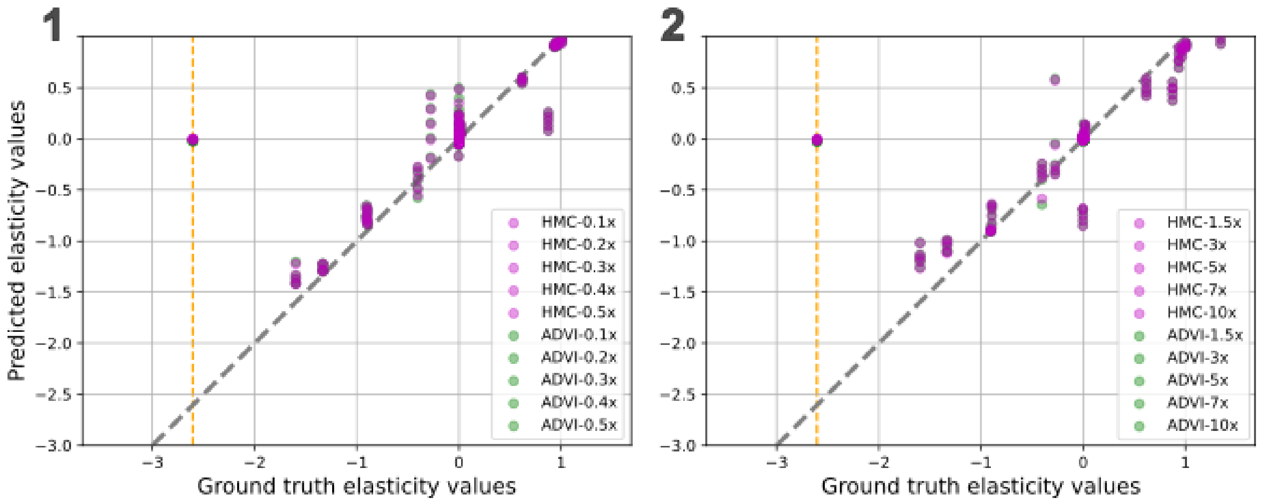

(Fig G) Comparison of ADVI and HMC for Topology B with one allosteric regulator (n=3). Both inference engines yield similar posterior elasticity estimates, failing to recover the ground-truth allosteric elasticity value (intersection of orange dashed line and gray dashed line). Panels show a zoomed-in region around the ground-truth value: 1) perturbations < 1x and 2) perturbations > 1x. This result indicates that the challenge in recovering strong allostery arises from the model formulation rather than the inference method.

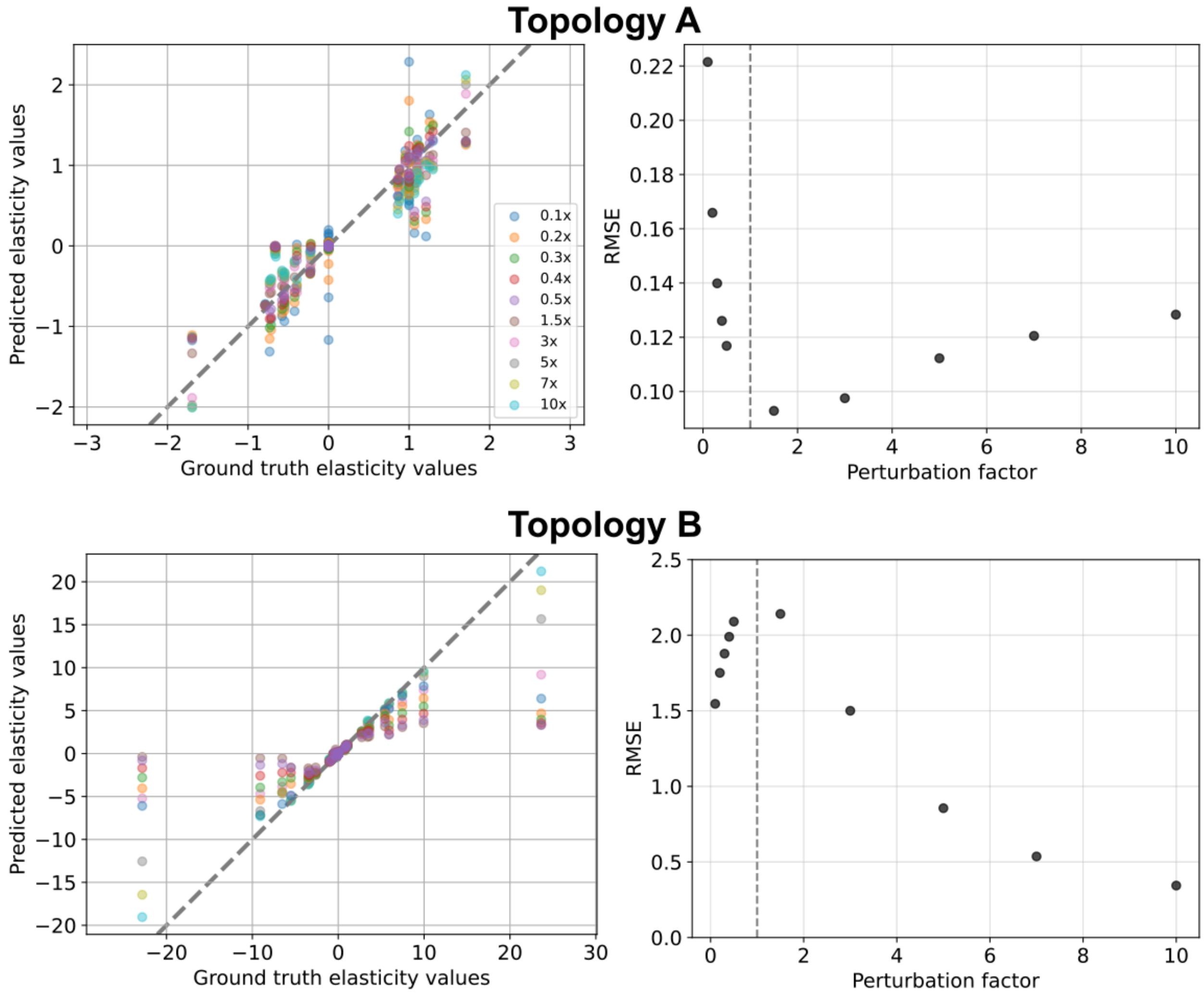

(Fig H) Influence of perturbation strength on elasticity predictions. Top) Topology A with two allosteric regulators and no data omitted. Bottom) Topology B with two allosteric regulators and no data omitted. Left: Elasticity predictions across different perturbation strengths. The color legend for perturbation strength is consistent across both plots. Right: Root mean square error (RMSE) of elasticity predictions at each perturbation strength. The dotted line shows the estimated RMSE between the measured perturbation strengths.The dashed line represents where the data was not perturbed (perturbation factor is 1x).

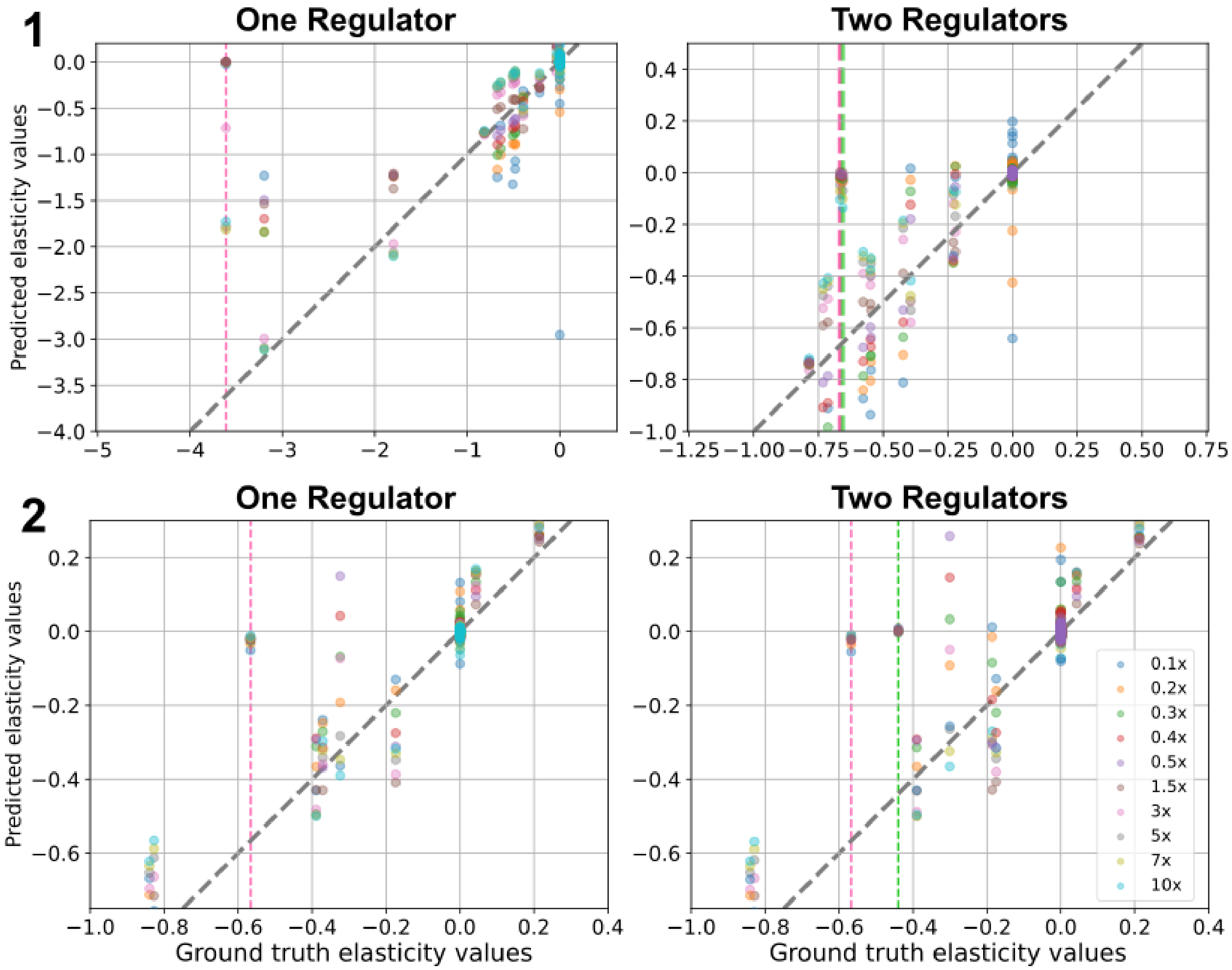

(Fig I) Elasticity predictions for model variations with weak allosteric regulators. BMCA-predicted elasticities from ten different perturbation strength datasets for 1) Topology A and 2) Topology B, each with one or two weak allosteric regulators (Hill coefficient = 1). Each dot represents the median of the predicted distribution means across the ten enzyme perturbation strengths. The pink dashed line indicates the ground truth elasticity of the first regulator, and the green dashed line indicates that of the second regulator. These graphs only show the elasticities in the vicinity of the ground truth elasticity value of the allosteric regulator.

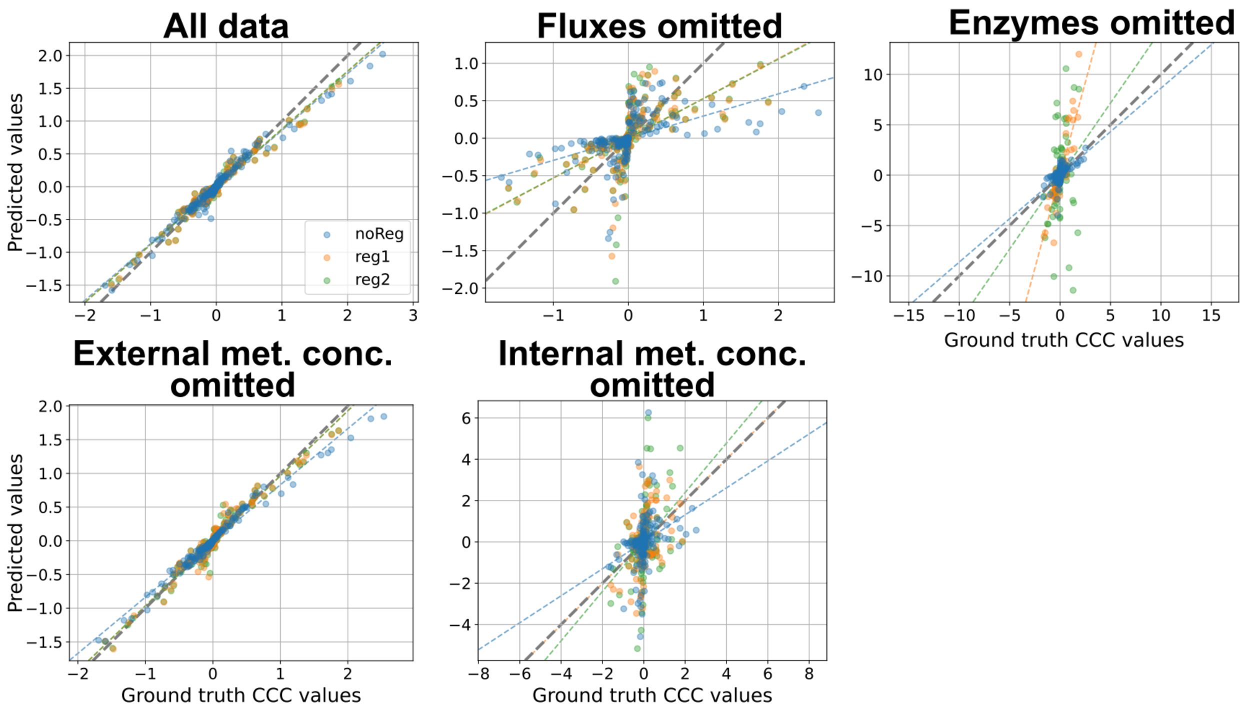

(Fig J) BMCA CCC predictions compared against ground truth values for Topology B. Each dot signifies the median of the predicted CCC values for the different enzyme perturbation levels tested. The titles for each graph indicate which data type was omitted when running BMCA.

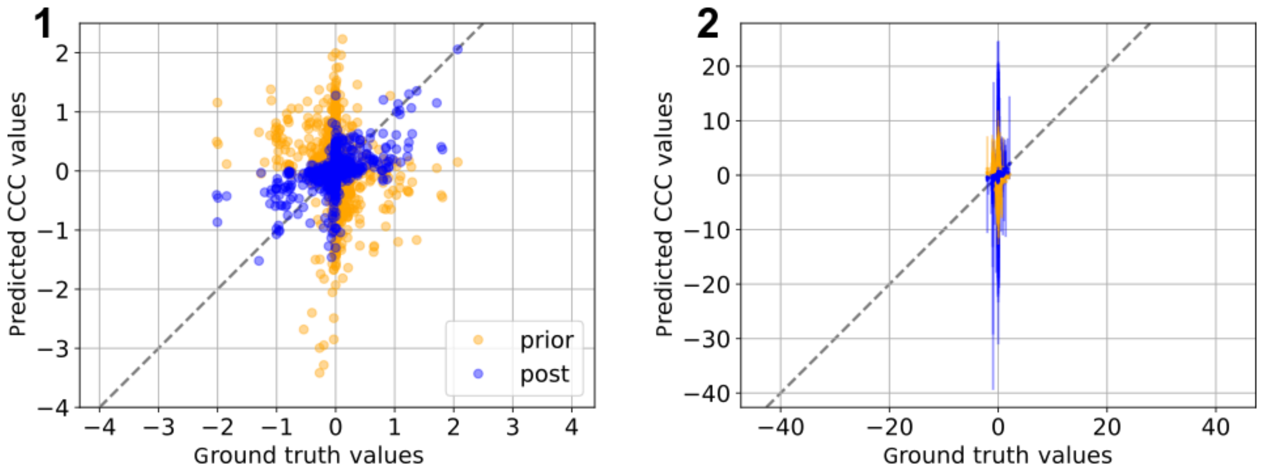

(Fig K) CCC value comparisons for Topology C. 1) Median of CCC values calculated from all perturbations of HDI elasticity distributions predicted by BMCA. 2) Ranges of possible values from the different perturbations.

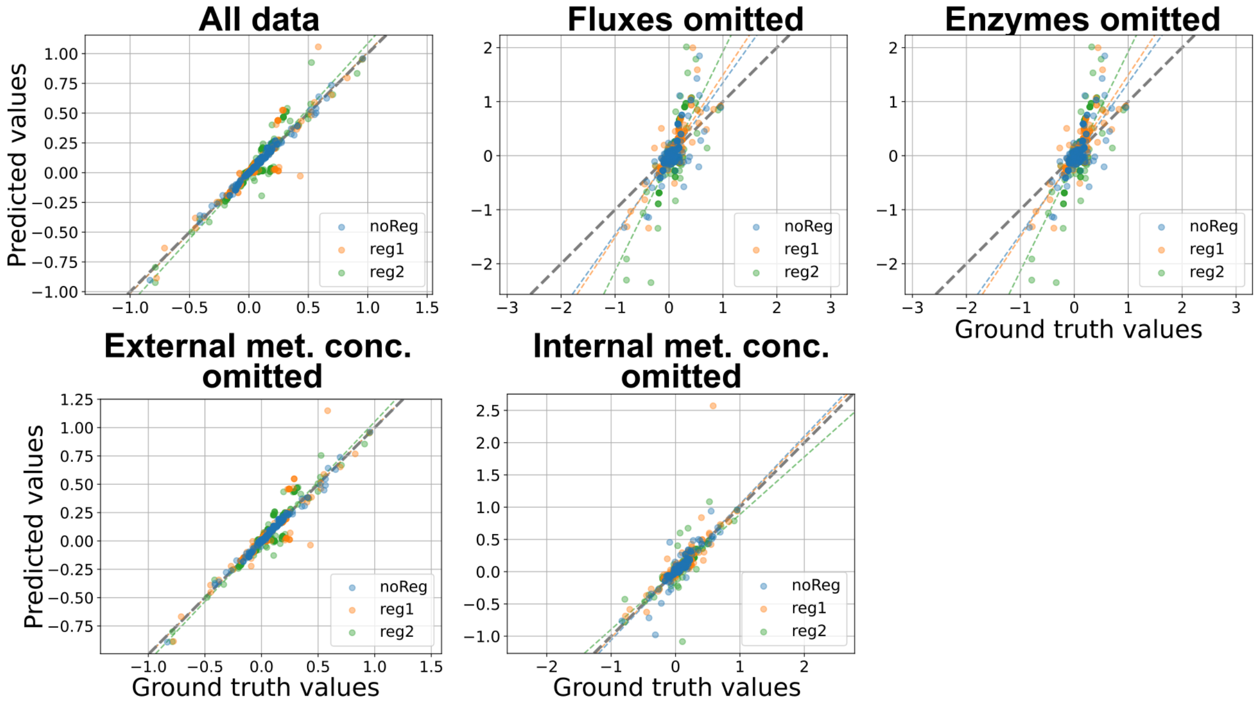

(Fig L) BMCA FCC predictions compared against ground truth values for Topology A. Each dot signifies the median of the predicted elasticity values across the different enzyme perturbation levels tested. The titles for each graph indicate which data type was omitted when running BMCA. Corrections for FCC values for reactions whose enzymes are being perturbed have been applied. The gray diagonal line indicates where the predicted values concur with the ground truth values.

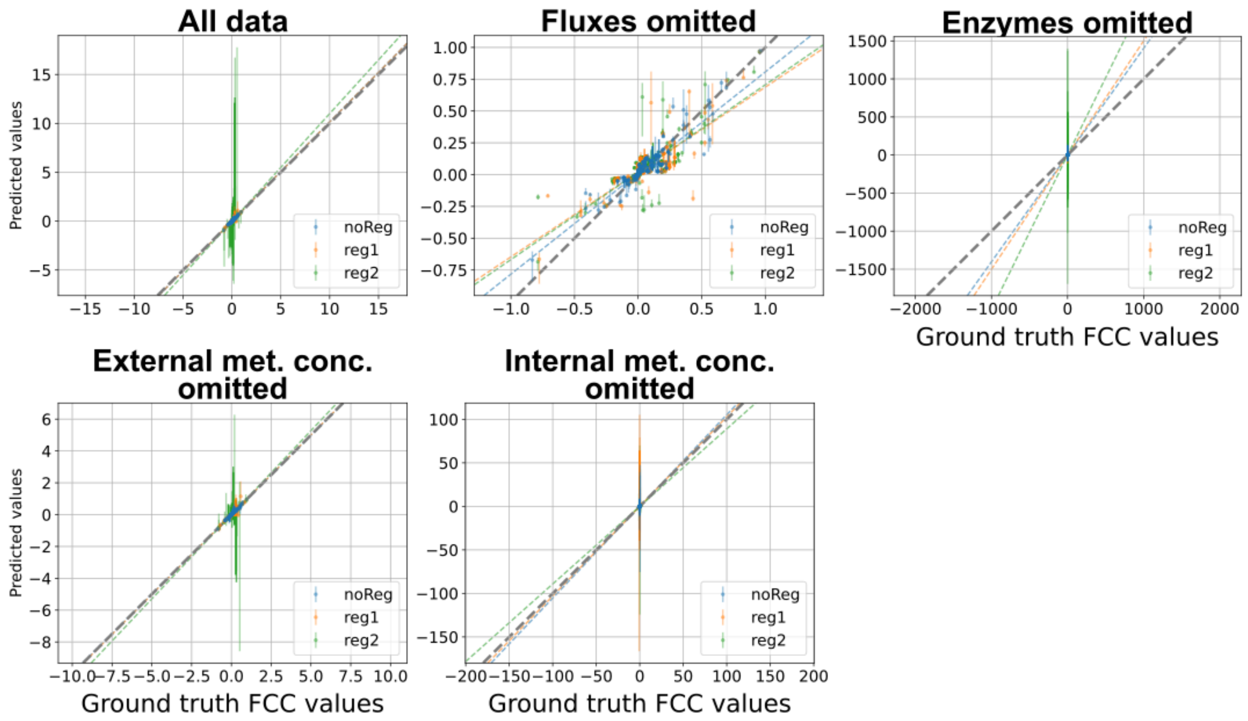

(Fig M) Uncertainty ranges for BMCA FCC predictions compared against ground truth values for Topology A. Each dot signifies the median while the error bars represent the range of the predicted elasticity values across the different enzyme perturbation levels tested. The titles for each graph indicate which data type was omitted when running BMCA. Corrections for FCC values for reactions whose enzymes are being perturbed have been applied. The gray diagonal line indicates where the predicted values concur with the ground truth values.

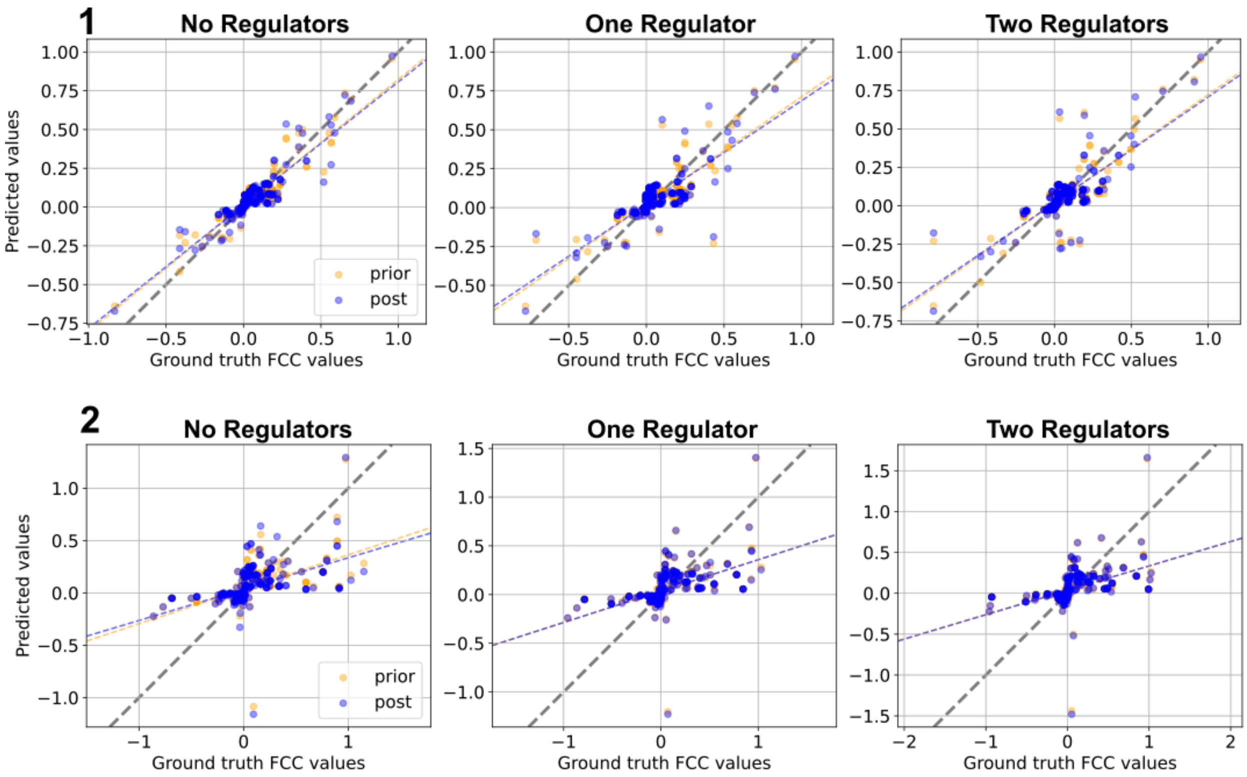

(Fig N) BMCA FCC predictions compared against ground truth values for Topology A and B model variations when flux values were omitted. Each row of graphs depicts the FCC values prior and post BMCA while varying the number of regulators in 1) Topology A and 2) Topology B. Each dot signifies the median while the error bars represent the range of the predicted elasticity values across the different enzyme perturbation levels tested. The titles for each graph indicate which data type was omitted when running BMCA. Corrections for FCC values for reactions whose enzymes are being perturbed have been applied. The gray diagonal line indicates where the predicted values concur with the ground truth values.

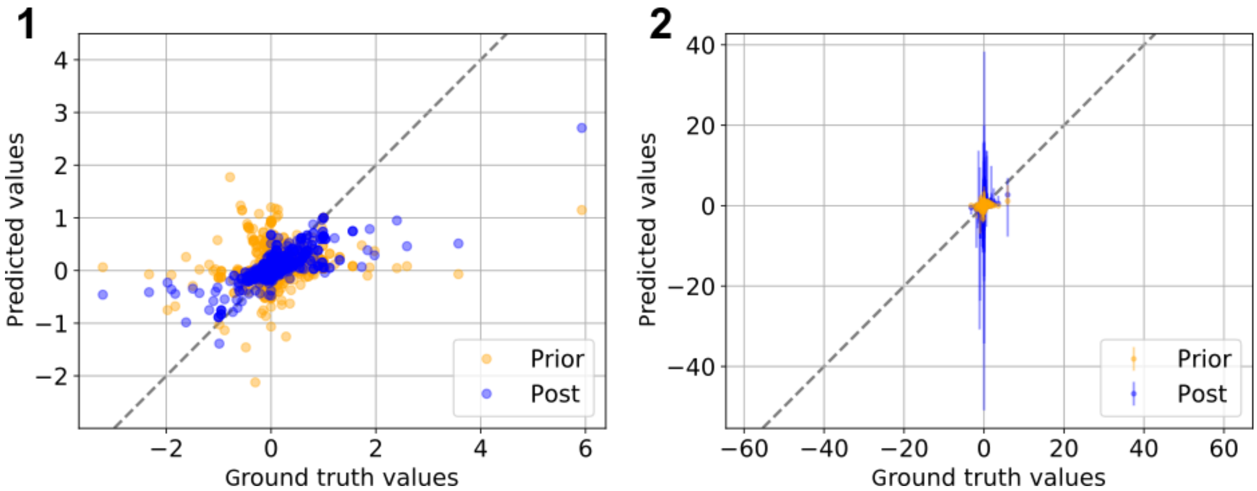

(Fig O) Topology C FCC value comparisons. 1) Median of FCC values calculated from all perturbations of HDI elasticity distributions predicted by BMCA. 2) Ranges of predicted FCC values across all perturbation strengths.

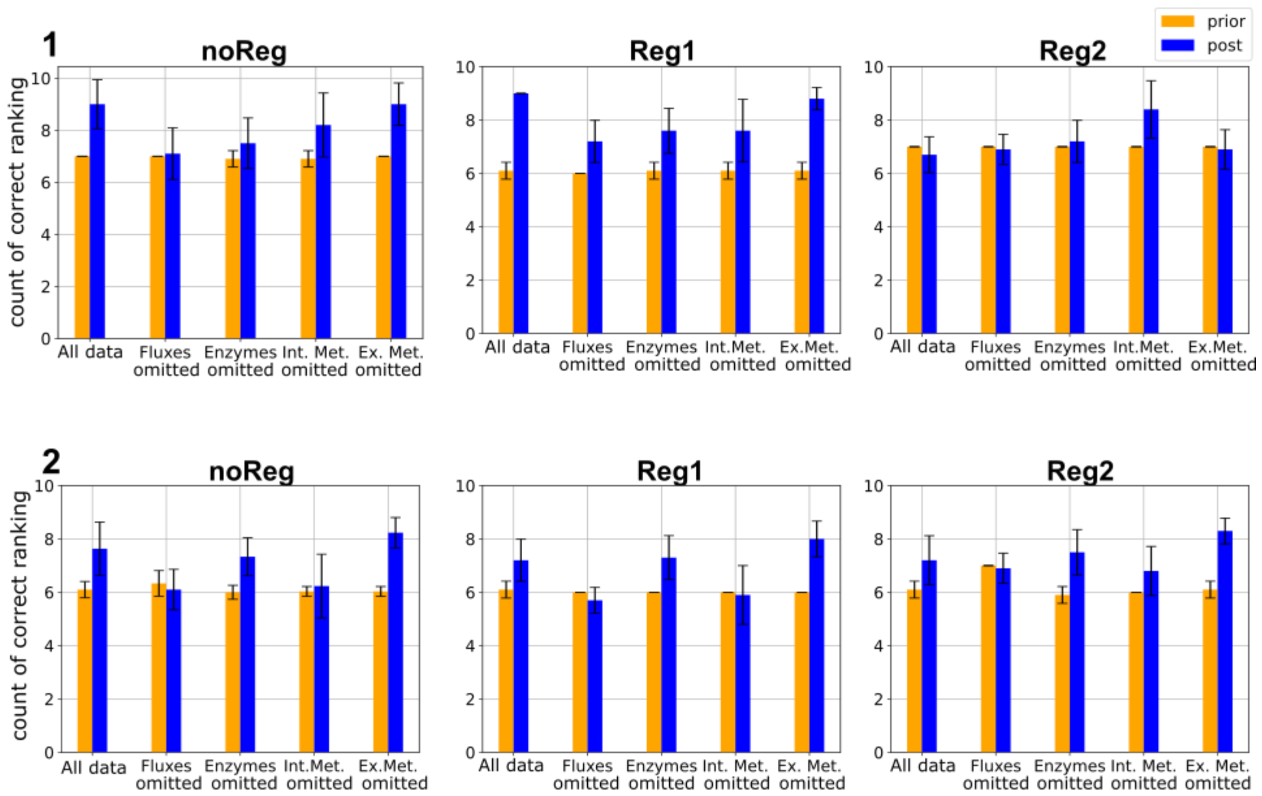

(Fig P) Number of enzymes correctly predicted as having one of the top ten highest FCC values based on the various data types omitted when running BMCA across different amounts of regulators. 1) Topology A, 2) Topology B. The height of the bars represent the mean of the predictions across all the perturbation levels and allosteric regulation levels; the error bars represent the standard deviation.

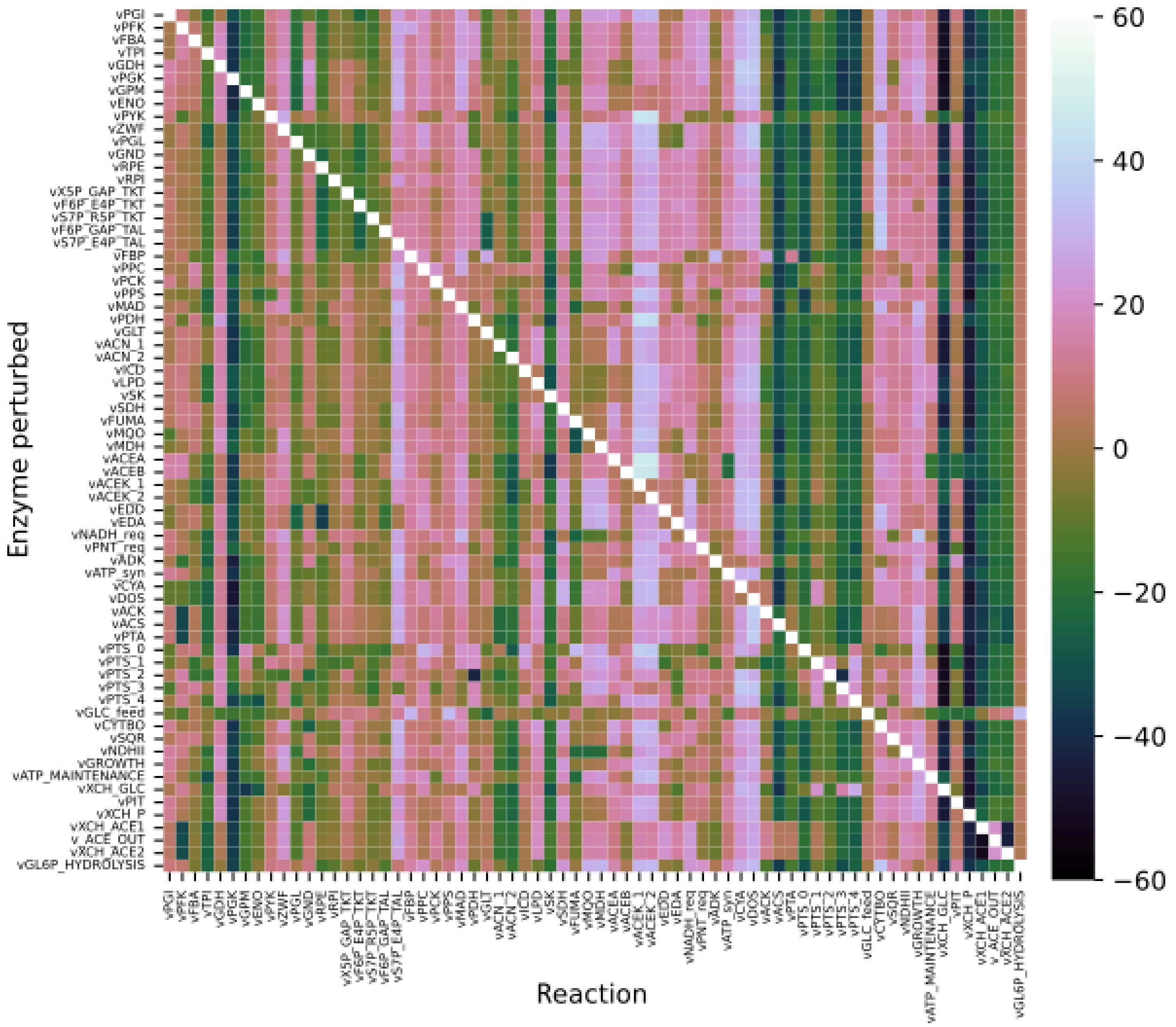

(Fig Q) Accuracy of FCC rankings predicted by the BMCA algorithm for Topology C. Y-axis describes which enzymes were perturbed while the x-axis describes the FCC being observed. The colorbar quantifies the difference in predicted ranking from the ground truth ranking.

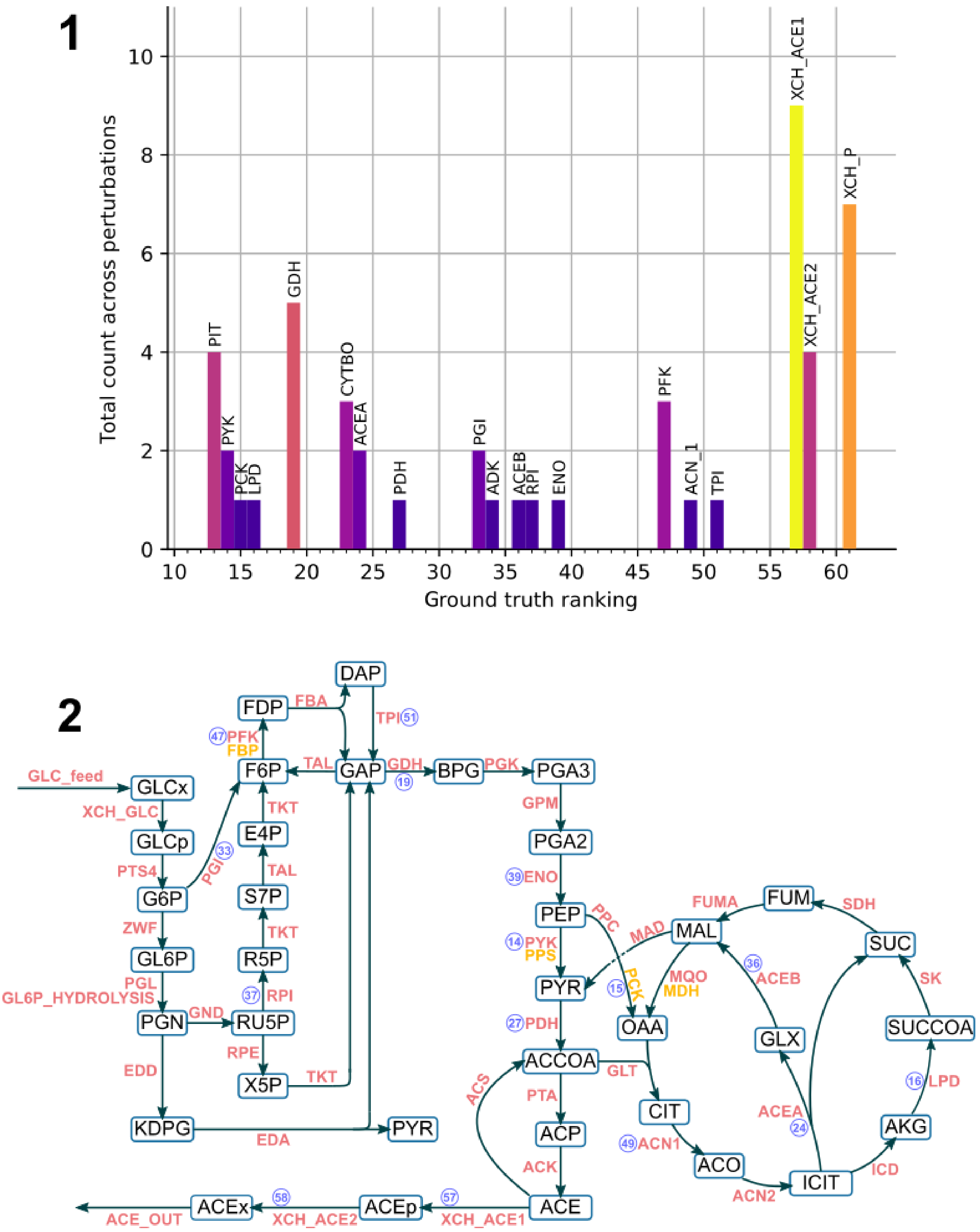

(Fig R) Ground truth rankings of enzymes that were incorrectly predicted as having a top ten highest FCC value for Topology C. 1) The bars represent the number of times an enzyme with a non-top ten ground truth ranking was incorrectly predicted across a total of ten different

perturbations. 2) Ground truth rankings shown in purple next to the reactions that were incorrectly predicted as being in the top 10. Reactions consisting of cofactors only are not shown.

Tables

	Data supplied	Mean	SD
**TopA-noReg**	All Data	9	1.05
	Omit Fluxes	6	0.00
	Omit Enzymes	7.1	0.74
	Omit Int. Met.	8.3	1.42
	Omit Ex. Met.	9.4	0.52
**TopA-Reg1**	All Data	8.9	0.32
	Omit Fluxes	7	0.00
	Omit Enzymes	7.3	1.25
	Omit Int. Met.	8	1.05
	Omit Ex. Met.	8.9	0.32
**TopA-Reg2**	All Data	6.8	0.79
	Omit Fluxes	8	0.00
	Omit Enzymes	7.2	0.63
	Omit Int. Met.	7.6	0.84
	Omit Ex. Met.	6.9	0.74
**TopB-noReg**	All Data	7.7	0.82
	Omit Fluxes	6	0.00
	Omit Enzymes	6.3	1.49
	Omit Int. Met.	6.2	1.03
	Omit Ex. Met.	8.1	0.88
**TopB-Reg1**	All Data	7.7	0.67
	Omit Fluxes	6	0.00
	Omit Enzymes	7.3	0.82
	Omit Int. Met.	6.4	0.84
	Omit Ex. Met.	7.7	0.82
**TopB-Reg2**	All Data	8.1	0.88
	Omit Fluxes	5	0.00
	Omit Enzymes	7.4	0.84

	Omit Int. Met.	6.2	1.40
	Omit Ex. Met.	8.6	0.70

(Table A) Top-10 overlap between predicted and ground truth FCC rankings. Values are reported as mean ± SD across replicates for each omission condition and topology.
